# Unveiling promising breast cancer biomarkers: an integrative approach combining bioinformatics analysis and experimental verification

**DOI:** 10.1186/s12885-024-11913-7

**Published:** 2024-01-31

**Authors:** Ali Golestan, Ahmad Tahmasebi, Nafiseh Maghsoodi, Seyed Nooreddin Faraji, Cambyz Irajie, Amin Ramezani

**Affiliations:** 1 Department of Medical Biotechnology, School of Advanced Medical Sciences and Technologies, Shiraz University of Medical Sciences, Shiraz, Iran; 2 Shiraz Institute for Cancer Research, School of Medicine, Shiraz University of Medical Sciences, Shiraz, Iran; 3 Institute of Biotechnology, Shiraz University, Shiraz, Iran; 4 Department of Pathology, School of Medicine, Shiraz University of Medical Sciences, Shiraz, Iran

**Keywords:** Breast cancer, Biomarker identification, Bioinformatic analysis, Differentially expressed genes, qRT-PCR

## Abstract

**Background:**

Breast cancer remains a significant health challenge worldwide, necessitating the identification of reliable biomarkers for early detection, accurate prognosis, and targeted therapy.

**Materials and methods:**

Breast cancer RNA expression data from the TCGA database were analyzed to identify differentially expressed genes (DEGs). The top 500 up-regulated DEGs were selected for further investigation using random forest analysis to identify important genes. These genes were evaluated based on their potential as diagnostic biomarkers, their overexpression in breast cancer tissues, and their low median expression in normal female tissues. Various validation methods, including online tools and quantitative Real-Time PCR (qRT-PCR), were used to confirm the potential of the identified genes as breast cancer biomarkers.

**Results:**

The study identified four overexpressed genes (*CACNG4*, *PKMYT1*, *EPYC*, and *CHRNA6*) among 100 genes with higher importance scores. qRT-PCR analysis confirmed the significant upregulation of these genes in breast cancer patients compared to normal samples.

**Conclusions:**

These findings suggest that *CACNG4*, *PKMYT1*, *EPYC*, and *CHRNA6* may serve as valuable biomarkers for breast cancer diagnosis, and *PKMYT1* may also have prognostic significance. Furthermore, *CACNG4*, *CHRNA6*, and *PKMYT1* show promise as potential therapeutic targets. These findings have the potential to advance diagnostic methods and therapeutic approaches for breast cancer.

**Supplementary Information:**

The online version contains supplementary material available at 10.1186/s12885-024-11913-7.

## Introduction

Breast cancer has become the most common cancer in women, surpassing lung cancer as the leading cause of cancer incidence. The high incidence rate of the disease, with more than 2.3 million new cases each year, continues to be a cause for concern [1]. Due to the variation in molecular traits, histological features, and clinical outcomes, breast cancer is classified into several subtypes, providing valuable insights into the disease and aiding treatment planning. Breast cancer is typically divided into six subgroups based on their molecular characteristics: basal-like, claudin-low, normal-like, luminal A and B, and HER2-positive. These subgroups have unique molecular profiles that distinguish their characteristics. The basal-like and claudin-low subtypes of triple-negative breast cancer (TNBC) lack expression of estrogen receptor (ER), progesterone receptor (PR), and HER2. These subtypes are associated with a higher risk of disease relapse and a greater likelihood of developing visceral metastases [2].

Biomarkers play a crucial role in identifying and predicting outcomes as well as therapeutic approaches for breast cancer. However, some commonly used biomarkers, including carcinoembryonic antigen (CEA), CA 15-3, and CA 27–29, have insufficient sensitivity and specificity, making them unsuitable for detecting breast cancer. They are recommended for monitoring disease progression and evaluating treatment response, particularly in patients with metastatic breast cancer [3]. On the other hand, biomarkers such as ER, PR, and HER2 have been extensively used in the management of breast cancer. They provide valuable information for prognosis and serve as targets for targeted therapy and hormone therapy [4]. In the pursuit of advancing breast cancer diagnosis and treatment, it is crucial for researchers to gain a comprehensive understanding of the molecular pathways that underlie breast carcinogenesis. Despite years of dedicated research into breast cancer patients, the overall 5-year survival rate remains unsatisfactory [5]. Consequently, there is a significant need for the discovery of reliable and novel biomarkers to aid in the early detection of breast cancer, enhance prognostic accuracy, enable precise prediction of disease behavior, and facilitate the development of targeted therapeutic approaches.

High throughput gene expression technologies provide comprehensive genetic information on cancer samples and identify changes in disease progression [6–8]. High throughput data like genomics, epigenomics, and transcriptomics in online databases were mined to identify potentially novel cancer-associated biomarkers. Recently, machine learning models such as support vector machine (SVM) and random forest have become attractive strategies for obtaining gene signatures.

The study identified new genes associated with breast cancer using large-scale transcriptomics data and the random forest technique. The expression of these genes in breast cancer tissues was validated using qRT-PCR and compared to normal tissues.

## Materials and methods

### Data collection and differential expression analysis

The RNA expression data for breast cancer was obtained from TCGA using the TCGA biolinks package [7]. Then, differential expression analysis between breast cancer and normal samples was performed using the edgeR Bioconductor package [7]. The DEGs (differentially expressed genes) were identified based on absolute fold changes > 2 and a false discovery rate (FDR) < 0.01. The top 500 up-regulated genes were selected from DEGs for further scrutiny and analysis.

To investigate the altered expression of selected genes, we investigated their expression levels in the Molecular Taxonomy of Breast Cancer International Consortium (METABRIC) database[9].

### Screening of important genes based on the random forest method

A random forest analysis was conducted to identify key genes. The expression data obtained from the TCGA database were initially normalized through log2 transformed fragments per kilobase of transcript per million mapped reads (UQ-FPKMs). Feature selection was then performed using the random forest classifier in the R package 'randomForest' to identify the most important gene features among the up-regulated DEGs. The random forest model's Gini index was used to discriminate between normal and cancer samples [10]. A higher Gini index value indicates greater relevance and importance of the gene in the classification process. Finally, the genes were ranked based on their significance level, and the top 100 genes with the highest Gini index values were selected as candidate feature genes for further investigation.

### Expression profiling analysis

The online database GEPIA2 (http://gepia2.cancer-pku.cn/#index) is a valuable resource that provides data derived from the Genotype-Tissue Expression (GTEx) and the TCGA databases. This database contains a comprehensive collection of RNA sequencing data for both cancer and normal tissues [11]. The GEPIA2 database was used to determine the expression levels of selected genes and their profiles based on pathological stages. Overexpressed genes in breast cancer tissues were identified in comparison to normal tissues. The UCSC Xena (http://xena.ucsc.edu) platform [12] and the UALCAN web resource (https://ualcan.path.uab.edu/) [13] were also used to compare tumor samples derived from TCGA and normal samples to validate the upregulation of selected genes in the breast cancer sample types.

### Subcellular localization study

GeneCards (https://www.genecards.org/) is a comprehensive and integrative database that provides information on all predicted and known human genes, including concise genomic, transcriptomic, proteomic, genetic, and functional information [14]. The GeneCards database was employed to undertake an initial evaluation of the subcellular location of each protein.

### Clinico-pathological variables associated with selected genes

Breast Cancer Gene-Expression Miner v4.8 analysis (bc-GenExMiner v4.8) was employed to assess the association between the expression pattern of selected genes and various clinico-pathological variables in breast cancer. These variables included Scarff-Bloom and Richardson grade status (SBR1, SBR2, and SBR3), BRCA1/2 status (Wild type and Mutated), and PAM50 subtypes (Basal-like, HER-2, Luminal A, Luminal B, and Normal breast-like) [15]. To determine statistically significant differences between groups, we employed Welch's test followed by the Dunnett-Tukey-Kramer's test. We considered a p-value of less than 0.05 as significant. The UALCAN web resource (https://ualcan.path.uab.edu/) [13] was used to assess further clinico-pathological features, including nodal metastasis status, TP53 mutation status, and the patient’s gender. The student’s t-test was employed to assess the differences in transcriptional expression.

### Functional enrichment analysis

GEPIA2 was used to identify genes with similar expression patterns, ranked by Pearson correlation coefficient (PCC). This facilitated the identification of closely related genes [9]. In addition, cBioPortal was used to identify genes positively associated with our selected genes through co-expression network analysis. The FunRich tool 3.1.3 [16] was then used to perform Gene Ontology (GO) and biological pathway enrichment analyses on the overlapping genes obtained from the GEPIA2 and cBioPortal databases. Furthermore, we conducted gene set enrichment analysis (GSEA) utilizing the GSEA software to investigate hallmark gene sets showing significant enrichment [17]. The expression levels of shared genes sourced from the GEPIA2 and cBioPortal databases were employed to assess the correlation between a gene set and a specific phenotype.

### Genetic alteration and somatic mutation analysis

The cBioPortal database (https://www.cbioportal.org/) was used to assess the genetic alterations of the selected genes [9]. The spectrum of genomic alterations, including mutations and putative copy-number alterations (CNAs), was analysed using default parameters and the GISTIC (Genomic Identification of Significant Targets in Cancer) algorithm. Additionally, the COSMIC database (cancer.sanger.ac.uk) [18] was employed to investigate the somatic mutations in the candidate genes.

### Survival analysis

The association between mRNA expression levels of the selected genes and overall survival (OS) outcomes in breast cancer patients was investigated using the Kaplan-Meier (KM) plotter database (https://kmplot.com/analysis/) [19]. Statistical significance in the analysis was determined using a log-rank p-value threshold of less than 0.05.

### Assessment of selected genes as potential therapeutic targets

To assess the potential impact of the selected genes on breast cancer cell growth and survival and to explore their suitability as potential therapeutic targets, the Cancer Dependency Map (https://depmap.org/portal/) was used [20].

### In vitro mRNA expression quantification

#### Tissue samples preparation

Fifty-five female breast cancer patients were included in this study, conducted at MRI Hospital in Shiraz, Iran, between 2015 and 2019. Patients were selected based on molecular pathology tests, biopsy results, and imaging techniques. Surgical procedures were performed to obtain samples of breast cancer tissues (BCT) as well as non-tumoral adjacent tissues (NTAT), serving as the normal control. The collected tissues were promptly frozen and stored at -70°C. Before tissue collection, none of the patients had undergone any form of treatment. Careful collection of tumor tissues from non-necrotic areas ensured that over 90% of the samples were of high-quality. The research protocol received ethical approval from the Research Ethics Committee of Shiraz University of Medical Sciences (Approval ID: IR.SUMS.REC.1401.215).

#### RNA extraction and cDNA synthesis

The RNX-Plus buffer (CinnaGen, Iran) was used to extract total RNA from snap-frozen tissues, following the manufacturer's instructions. To prevent potential contamination with genomic DNA, DNase I treatment was applied during the RNA extraction process. Afterward, cDNA synthesis was conducted using a cDNA synthesis kit (Fermentas, Lithuania), which combined oligo-dT primers and random hexamers.

#### qRT-PCR

The ABI StepOne instrument (Applied Biosystems, USA) was used to perform the qRT-PCR experiments in 48-well microtitre plates. Each reaction consisted of a total volume of 20 µL containing primers specific for the target genes and an ABI SYBR Green master mix. The amplification process included an initial denaturation step at 95 °C for 10 minutes, followed by 45 cycles of denaturation at 95 °C for 20 seconds, and annealing/extension at 60 °C for 60 seconds. To ensure accurate quantification, the CtNorms algorithm was applied to normalize amplification efficiency [21]. Melt curve analysis was performed to confirm the specificity of the qRT-PCR results. Data normalization was carried out using the 2^-ΔΔCT^ formula, a widely used method for comparing gene expression levels between different samples. Specific primers for the target genes (*CACNG4*, *PKMYT1*, *EPYC*, and *CHRNA6*) and the internal control gene (*Actin Beta*) were designed using Allele ID 7 software (see Table 1). Each sample was tested in triplicate, and the final result was determined by the average Ct value. To validate the qRT-PCR results and exclude the possibility of genomic DNA contamination, additional PCR reactions were conducted using extracted RNA samples without reverse transcription. This step was implemented to confirm that the PCR results originated from complementary DNA (cDNA) and not from genomic DNA.

**Table 1:** Primer sequences

Gene symbol	Primer sequences
***CACNG4***	Forward: 5´-CAATGACTACGACCACGACAG-3´ Reverse: 5´-GCAGCCACGAAGAGGATG-3´
***PKMYT1***	Forward: 5´-GCCAGAGTCCTTCTTCCAG-3´ Reverse: 5´-GAACGCTTTACCGCATAGAG-3´
***EPYC***	Forward: 5´-CCAGGAAGAGGAAGAGGAGGAGGAAT-3´ Reverse: 5´-GGCAGCGGAGGAATAGCATCAAGT-3´
***CHRNA6***	Forward: 5´- TCACAGAAACCATCCCATCCACAT -3´ Reverse: 5´- TCAACACAAACACAGTCACCACG -3´
***ACTB***	Forward: 5´-GCCTTTGCCGATCCGC-3´ Reverse: 5´-GCCGTAGCCGTTGTCG-3´

Primer sequences were designed via Allele ID 7 software for the qRT-PCR technique with an annealing temperature of 60 °C

### Clinico-pathological data collection

Clinico-pathological data, including age, human epidermal growth factor receptor 2 (HER2) status, progesterone receptor (PR) status, estrogen receptor (ER) status, lymph nodes (LN) involvement, and molecular breast cancer subtypes (luminal, HER2 overexpressed, and TNBC) were collected from the patient's medical records. The data were then compiled and analyzed to assess their association with *CACNG4*, *PKMYT1*, *EPYC*, and *CHRNA6* gene expression patterns in the breast cancer tissue samples. This step was important in determining the potential clinical relevance of these genes as biomarkers for breast cancer diagnosis and prognosis. At last, to assess the potential correlation between these gene expression patterns, a nonparametric Spearman correlation coefficient was calculated using the expression data obtained from the qRT-PCR analysis.

### Statistical analysis

The data was analyzed using GraphPad Prism 9.4.0 software (GraphPad Software, Inc., USA). A paired t-test was employed to determine the mean differences of the *CACNG4*, *PKMYT1*, *EPYC*, and *CHRNA6* genes between BCT and NTAT tissues. At the same time, the Mann-Whitney test was used to assess the normalized expression ratio concerning the clinico-pathological features of the study population. The Kruskal-Wallis test was also applied to examine the variations among breast cancer subtypes. The nonparametric Spearman correlation coefficient was used to measure the expression correlation between these genes (*CACNG4*, *PKMYT1*, *EPYC*, and *CHRNA6*).

## Results

### Screening of important genes

The RNA expression data were extracted from the TCGA database to compare the differentially expressed genes (DEGs) between breast invasive carcinoma (BRCA) and normal samples. A random forest algorithm was then used to determine the significance of the up-regulated DEGs and identify important gene features (Additional files 1 & 2). After the selection of 100 genes with higher importance scores, these genes were subjected to a meticulous selection process. Genes exhibiting overexpression in breast cancer tissues compared to normal tissues were identified through an evaluation utilizing UCSC Xena server, UALCAN, and GEPIA2 databases. Then GEPIA2 was employed to discriminate genes with a low median expression in normal female tissues. Finally, a comprehensive examination and analysis of relevant scientific literature and articles were conducted to identify a novel panel of potential diagnostic biomarkers among the determined DEGs, highlighting the innovative aspects of gene exploration. Through this screening process, four genes, namely *CACNG4*, *PKMYT1*, *EPYC*, and *CHRNA6*, were identified, and then the prognostic and therapeutic implications of these selected genes were investigated using various databases. Based on METABRIC database, *CACNG4*, *PKMYT1*, and *CHRNA6* exhibited differential expression in breast cancer tissues compared to normal tissues. However, *EPYC* did not display such a distinction (Additional file 3).

### Profiles of mRNA expression

**Fig. 1:**
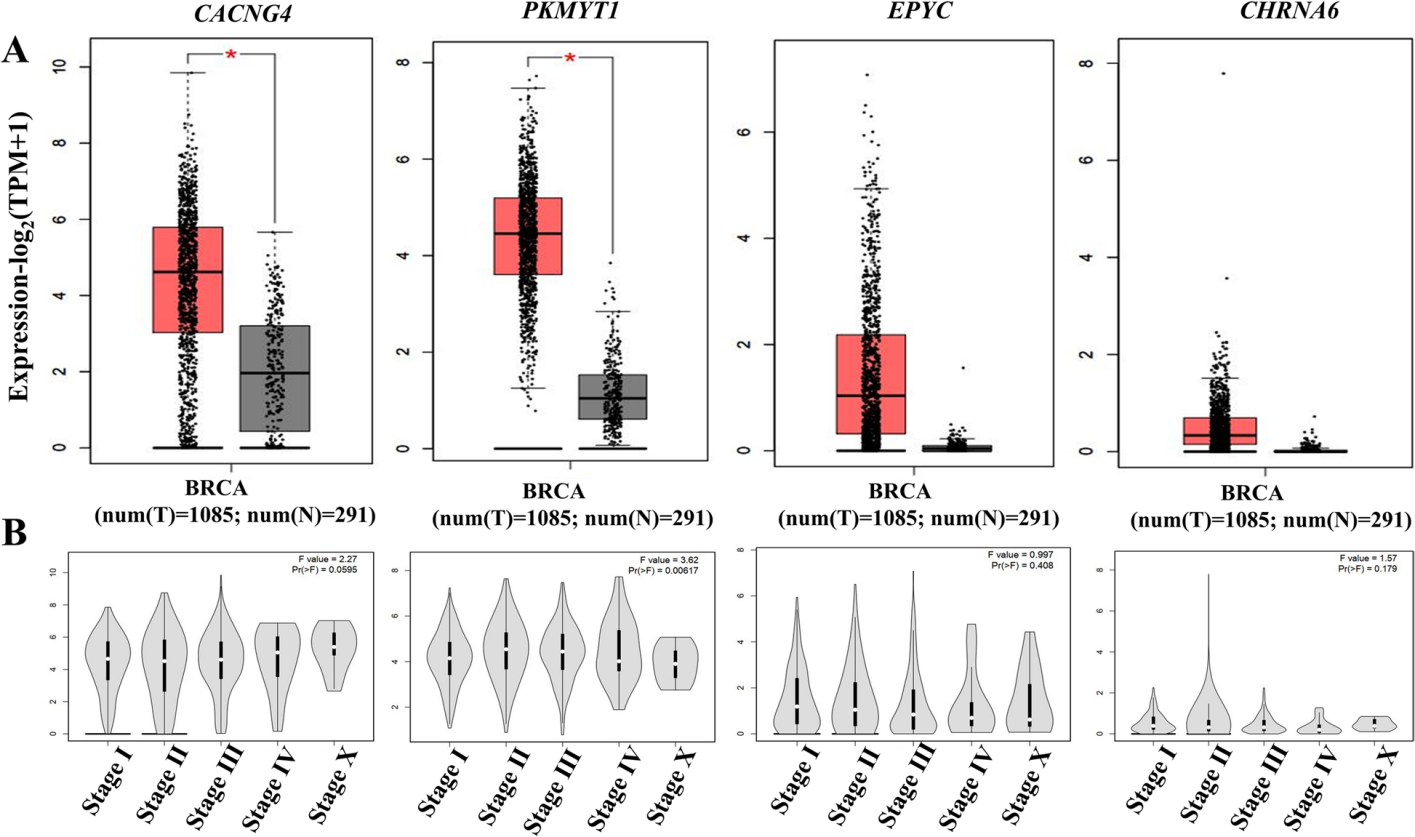
Expression analysis and stage correlation in Breast Invasive Carcinoma (BRCA) patients from GEPIA2 Database. **A** Expression level of *CACNG4*, *PKMYT1*, *EPYC*, and *CHRNA6* between BRCA and normal breast tissues. **B** Correlation with Tumor Stages. TPM, transcripts per million

The GEPIA2 database was used to compare the expression levels of the selected genes between breast cancer patient tissues and normal subjects. The analysis revealed an overexpression of *CACNG4*, *PKMYT1*, *EPYC*, and *CHRNA6* genes in BRCA tissues compared to normal breast tissues, while this overexpression was statistically significant in the case of *CACNG4* and *PKMYT1* genes, as depicted in Fig. 1A. The differential expression of these four genes was also analyzed using the UCSC and UALCAN databases. Using the Xena UCSC tool and the UALCAN web resource (https://ualcan.path.uab.edu/), it was found that the expression levels of all four selected genes were significantly higher in breast cancer than in normal tissues, as presented in Additional file 4. In addition, the expression level of *PKMYT1* (*P* = 0.006) showed differential expression across tumor stages, whereas the expression levels of *CACNG4* (*P* = 0.06), *EPYC* (*P* = 0.4), and *CHRNA6* (*P* = 0.2) did not show any statistically significant differences, as illustrated in Fig. 1B.

### Prediction of subcellular localization

The GeneCards database was employed to determine the subcellular localization of the identified proteins. Considering the significance of cell membrane proteins as therapeutic targets, the identified genes were investigated for subcellular localization. CACNG4 and CHRNA6 were predicted to be localized to the plasma membrane. In contrast, PKMYT1 was predicted to be localized in the nucleus and cytosol, while EPYC was predicted to be located in the extracellular matrix (Additional file 5).

### Investigating the correlation of expression with clinico-pathological parameters

We investigated the expression level of *CACNG4*, *PKMYT1*, *EPYC*, and *CHRNA6* genes in breast cancer patients categorized by Scarff-Bloom and Richardson grade status (SBR1, SBR2, and SBR3), BRCA1/2 status (Wild type and Mutated), and PAM50 subtypes (Basal-like, HER-2, Luminal A, Luminal B, and Normal breast-like) using the Breast Cancer Gene-Expression Miner v4.8 databases (bc-GenExMiner v4.8) (see Additional files 6 & 7). The results revealed significant differences in the expression levels of *CACNG4*, *PKMYT1*, and *CHRNA6* among SBR1, SBR2, and SBR3 (*CACNG4* (SBR1 and SBR 2> SBR3, SBR1=SBR2) *PKMYT1* (SBR3>SBR2>SBR1) *CHRNA6* (SBR1 and SBR 2> SBR3, SBR1=SBR2)). However, no difference was observed in the expression of the *EPYC* gene among SBR1, SBR2, and SBR3, as displayed in Additional file 7 (Supplementary Fig. 4A). Moreover, when comparing BRCA1/2 status, no significant differences in the expression levels of *CACNG4* and *EPYC* were found across wild type and mutated BRCA1/2. In contrast, the expression level of *PKMYT1* in the mutated ones was higher than that of the wild type, and the expression level of *CHRNA6* in the mutated group was lower than that of the wild type as shown in Additional file 7 (Supplementary Fig. 4A). Furthermore, this analysis revealed various expressions of *CACNG4*, *PKMYT1*, *EPYC*, and *CHRNA6* in different BRCA subtypes compared to normal breast-like (*CACNG4* (HER-2, Luminal A, and B > normal breast-like, basal-like < normal breast-like), *PKMYT1* (basal-like, HER-2, and Luminal B > normal breast-like, Luminal B < normal breast-like), *EPYC* (Luminal A and B > normal breast-like, basal-like < normal breast-like HER-2 = normal breast-like), and *CHRNA6* (HER-2, Luminal A, and B, basal-like > normal breast-like)) that can be seen in Additional file 7 (Supplementary Fig. 4A). To supplement our findings, we analyzed *CACNG4*, *PKMYT1*, *EPYC*, and *CHRNA6* genes expression and clinico-pathological parameters based on nodal metastasis status (Normal, N0, N1, N2, N3), TP53 mutation status (Normal, TP53 mutant, and TP53 non-mutant), and patient’s gender (Normal cases, Male and Female patients) through UALCAN database. The findings revealed significant variations in the expression levels of *CACNG4*, *PKMYT1*, *EPYC*, and *CHRNA6* mRNA in nodal metastasis status (N0, N1, N2, N3 > Normal) and TP53 mutation status (TP53 mutant and TP53 non-mutant > Normal)) (Additional file 7, Supplementary Fig. 4B). Furthermore, an evaluation of the patient’s gender demonstrated that *CACNG4*, *PKMYT1*, and *CHRNA6* mRNA expression levels were significantly higher in male and female patients than in normal cases. Notably, the expression level of *EPYC* mRNA in female patients was higher than in normal cases (p < 0.05); however, there was no significant difference in the expression of *EPYC* mRNA between male patients and normal cases (p > 0.05) as illustrated in Additional file 7 (Supplementary Fig. 4B).

### Functional and pathway enrichment analysis

The GEPIA2 database (BRCA dataset) and cBioPortal dataset (TCGA, PanCancer Atlas) were used to select the genes co-expressing with *CACNG4*, *PKMYT1*, *EPYC,* and *CHRNA6* genes. Data from these two databases were crossed to identify the common genes. The co-expressed genes were subjected to gene ontology and pathway analysis using the FunRich tool (version 3.1.1) (Detailed data are supplied in the Additional files 8 to 12). GO enrichment analysis categorizes gene functions into three distinct groups: biological process (BP), molecular function (MF), and cellular component (CC). Based on GO analysis, the common co-expressed genes of *CACNG4* were considerably prominent in the subcategories of plasma membrane, transport activity, and signal transduction (Additional file 8).

Similarly, the *PKMYT1* common co-expressed genes were enriched in the nucleus, DNA binding, and cell cycle subcategories (Additional file 9). In contrast, the *EPYC* common co-expressed genes were enriched in the extracellular matrix, extracellular matrix consistent, and cell growth subcategories (Additional file 10). In addition, the *CHRNA6* common co-expressed genes were enriched in the subcategories of cytoplasm, transcription regulation activity, and cell communication subcategories (Additional file 11). Our biological pathway enrichment analysis revealed that *CACNG4* was significantly associated with the ErbB receptor signaling network and the mTOR signaling pathway. At the same time, *PKMYT1* was enriched in the DNA replication and cell cycle pathways. Furthermore, *EPYC* was mainly associated with epithelial-to-mesenchymal transition, and *CHRNA6* was observed to be involved in the ErbB receptor signaling network and signal transduction. We also conducted the GSEA to pinpoint the most notable hallmark gene sets. The results of the GSEA showed enrichment in 5 gene sets (Additional file 13).

### Genetic alterations and somatic mutations in selected genes

The cBioPortal database was utilized to investigate the frequency of genetic alterations in *CACNG4*, *PKMYT1*, *EPYC*, and *CHRNA6* genes in BRCA. Our findings indicate an overall alteration frequency of 37% across all queried genes, as shown in Additional file 14. Notably, the highest proportion (16%) of cases were patients with *CACNG4* alteration, resulting in mRNA upregulation. Conversely, for the *CHRNA6* gene, amplification was the most common alteration (6.55%). Furthermore, the COSMIC database analyzed the mutations of the *CACNG4*, *PKMYT1*, *EPYC*, and *CHRNA6* genes in breast cancer. Additional file 14 provides details on the types of mutations observed in these four genes. The most significant proportion of cases for all four genes were missense mutations among other types of transformation.

### Prognostic potential of selected genes

**Fig. 2:**
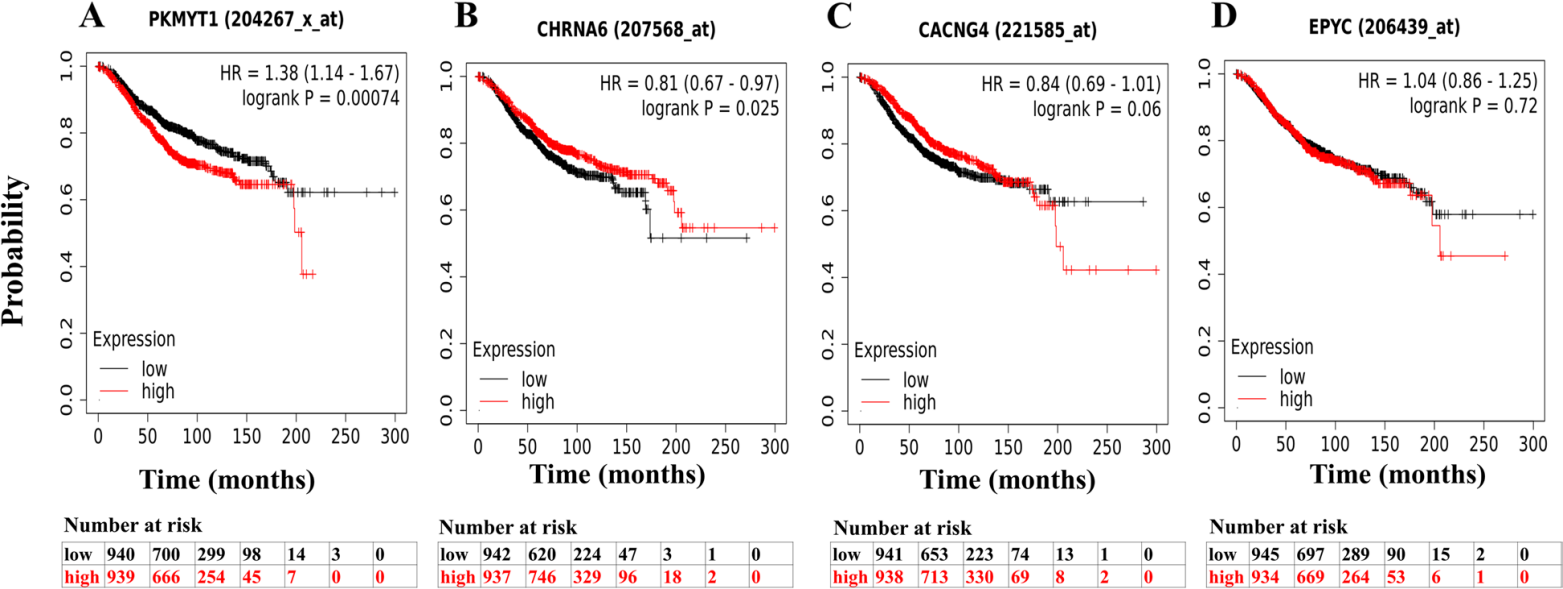
Survival analysis of query genes in BRCA patients from the Kaplan-Meier plotter database. Overall survival curves of (A) *PKMYT1*, (B) *CHRNA6*, (C) *CACNG4*, and (D) *EPYC* were analyzed. A log-rank *p*-value below the 0.05 threshold indicates a statistically significant association. HR, Hazard ratio

Based on the findings obtained from the Kaplan-Meier plotter database analysis, it was observed that a higher expression of *PKMYT1* exhibited a significant association with unfavorable overall survival (OS) outcomes in BRCA (OS Hazard Ratio (HR) = 1.38, log-rank p-value = 0.00074) as illustrated in Fig. 2A. In contrast, a high expression of *CHRNA6* was shown to confer a potentially favorable prognosis (OS HR=0.81, logrank *P*=0.025) (Fig. 2B). However, there was no statistically significant difference in OS for *CACNG4* (OS HR=0.84, logrank *P*=0.06) (Fig. 2C) and *EPYC* (OS HR=1.04, logrank *P*=0.72) genes (Fig. 2D).

### Assessment of tumor cell line dependency on CACNG4, PKMYT1, EPYC, and CHRNA6

Tumor cell growth and survival are dependent on the expression levels of some of the therapeutic biomarkers. The Cancer Dependency Map analysis tool is one of the databases that can help identify biomarkers associated with tumour cell viability. In this study, the DepMap tool was used to evaluate the significance of identified biomarkers for breast tumour cell growth and survival. Specifically, siRNA and CRISPR screening data were analyzed to determine the likelihood of breast cell line dependency on the identified genes, as indicated by the dependency scores. A lower Chronos score suggests a higher likelihood that the gene of interest is crucial in a particular cell line. Among our identified genes, *PKMYT1* exhibited a significant dependency score in the case of CRISPR knockout (a lower Chronos score), while *CACNG4*, *EPYC*, and *CHRNA6* did not display substantial dependency scores, as indicated in Additional file 15.

### mRNA expression quantification

**Fig. 3:**
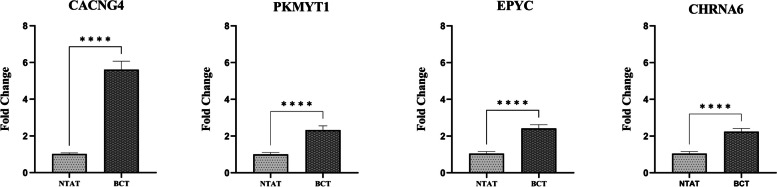
The relative gene expression levels of *CACNG4*, *PKMYT1*, *EPYC*, and *CHRNA6* were compared between non-tumoral adjacent tissues (NTAT) and breast cancer tissues (BCT) using the qRT-PCR technique. The findings revealed a significant upregulation of mRNA expression levels for *CACNG4*, *PKMYT1*, *EPYC*, and *CHRNA6* in BCT compared to NTAT (**** indicates a p-value less than 0.0001)

Based on qRT-PCR analysis conducted on 55 breast cancer patients and normal cases, it was observed that *CACNG4*, *PKMYT1*, *EPYC*, and *CHRNA6* mRNA exhibited significantly higher expression levels in breast cancer tissues (BCT) compared to non-tumoral adjacent tissues (NTAT) (*P* < 0.0001). As depicted in Fig. 3, paired t-tests comparing the gene expression profiles of *CACNG4*, *PKMYT1*, *EPYC*, and *CHRNA6* between breast tumors and normal tissues revealed an almost 5.55-fold, 2.31-fold, 2.32-fold, and 2.14-fold increase in breast tumors, compared to normal tissues, respectively. Notably, PCR reactions performed on extracted RNA samples without reverse transcription showed no amplification.

### Association of gene expression pattern with clinico-pathological characteristics

**Fig. 4:**
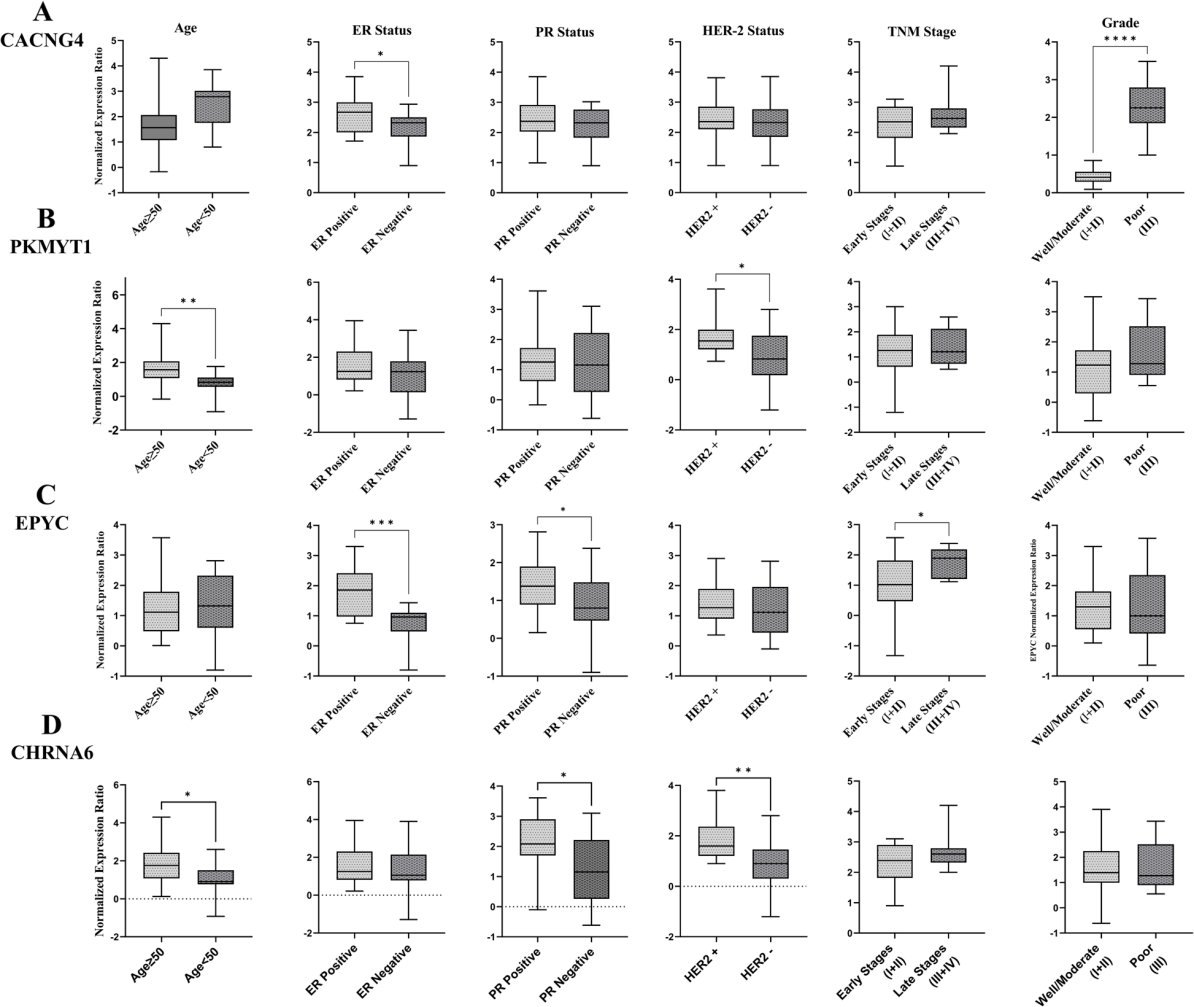
Association of gene expression patterns of *CACNG4*, *PKMYT1*, *EPYC*, and *CHRNA6* with clinico-pathological features in breast cancer patients. The expression patterns of these genes with age, ER, PR, and HER2 status, TNM stages, and histological grades. Statistical significance was indicated using asterisks (* *p* < 0.05, ** *p* < 0.01, and *** *p* < 0.001). ER: Estrogen Receptor; PR: Progesterone Receptor; HER2: Human Epidermal Growth Factor Receptor 2; and TNM: Tumor, Node, Metastasis

**Fig. 5:**
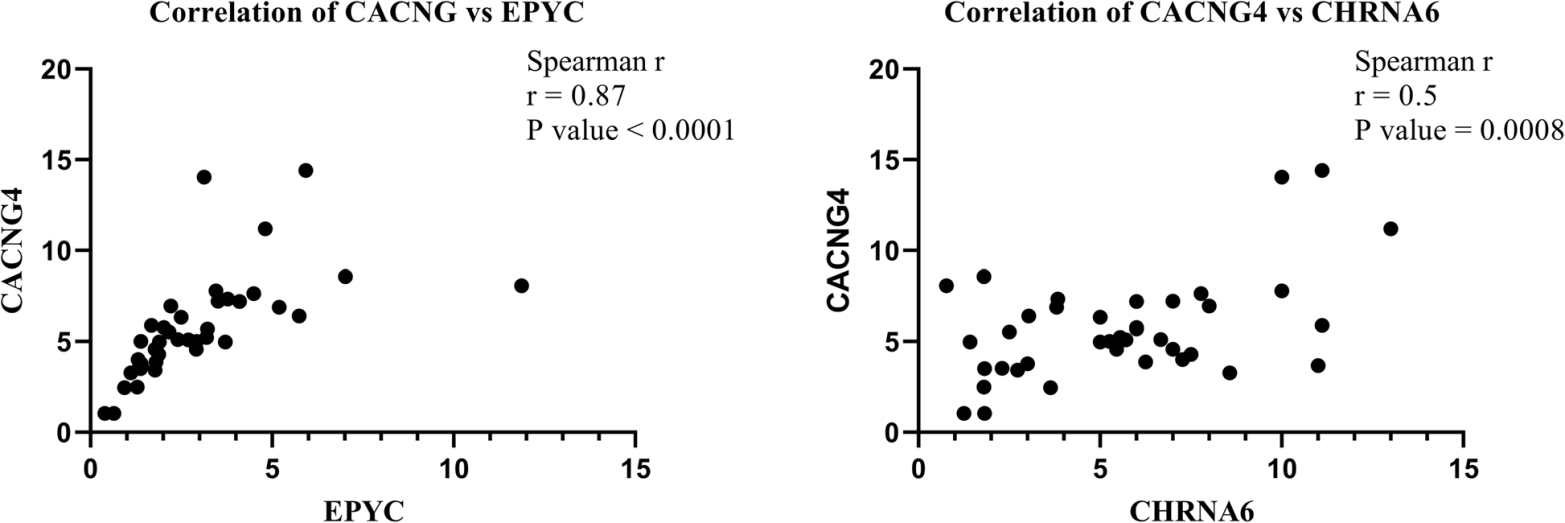
Correlation analysis. Spearman correlation analysis based on qRT-PCR results of 55 breast cancer patients showed a significant positive correlation between *CACNG4* and *EPYC* mRNA expression (Spearman's correlation = 0.87, *P* < 0.0001), and a positive correlation was observed between *CACNG4* and *CHRNA6* mRNA expression (Spearman's correlation = 0.5, *P* = 0.0008)

An evaluation of the clinico-pathological features of the breast cancer patients revealed that the mean age was 57.6 ± 11.5 (37-76) years. Most patients (77.5%) were diagnosed with early-stage breast cancer, specifically stages I and II, and 47% had positive lymph node involvement. Fig. 4 and Additional file 16 show the association between the relative expression of *CACNG4*, *PKMYT1*, *EPYC*, and *CHRNA6* genes with clinico-pathological features, including age, ER, PR, HER2 status, TNM stages, and histological grades, which were assessed using the Mann-Whitney test. Analysis of *CACNG4* mRNA expression unveiled a significant upregulation in patients with grade III breast cancer compared to patients with grade I and II tumors. Additionally, a significant increase in *CACNG4* mRNA expression was observed in ER-positive breast cancer patients compared to ER-negative cases, as illustrated in Fig. 4A. *PKMYT1* mRNA expression was up-regulated in patients aged 50 years and older, as well as in patients with HER2-positive status, as displayed in Fig. 4B. Analysis revealed a significant increase in *EPYC* mRNA expression levels in hormone receptor-positive (HR+) breast cancer patients compared to HR-negative (HR-) individuals. Moreover, a substantial upregulation of *EPYC* mRNA expression was observed in late-stage (III+IV) breast cancer patients compared to early-stage (I+II) patients, as depicted in Fig. 4C. Fig. 4D shows that *CHRNA6* mRNA expression was higher in patients over 50 years of age, as well as in patients with PR-positive and HER2-positive status. The association of these identified genes with lymph node status and breast cancer subtypes was also evaluated, but no significant differences were observed. Therefore, these findings were not included in Fig. 4. Nonparametric Spearman correlation analysis was conducted to assess the strength of association between the identified genes. A significant positive correlation was observed between *CACNG4* and *EPYC* mRNA expression levels (Spearman's correlation coefficient = 0.87, P value < 0.0001), and between *CACNG4* and *CHRNA6* mRNA expression levels (Spearman's correlation coefficient = 0.5, *P* value = 0.0008). However, no significant correlations were observed between *PKMYT1* and *EPYC*, *PKMYT1* and *CACNG4*, *PKMYT1* and *CHRNA6*, as well as *EPYC* and *CHRNA6* mRNA expression levels in breast cancer patients (Fig. 5).

## Discussion

There is an urgent need to characterize new biomarkers that can facilitate early detection of breast cancer and overcome the limitations of mammography and the challenges of current tumor biomarkers such as CA-125 and CEA [22, 23].

In this study, RNA expression data was obtained from TCGA to identify DEGs between BRCA and normal samples. The up-regulated genes were then analyzed using the random forest algorithm to identify the most important genes. These key genes were further investigated based on their overexpression in breast cancer tissues, low median expression in normal female tissues, and potential as novel diagnostic biomarkers. Four genes were identified from this screening: *CACNG4*, *PKMYT1*, *EPYC*, and *CHRNA6*. Integrated online bioinformatics databases were used to gain insight into the diagnostic, prognostic, and therapeutic roles of these identified potential biomarkers. Analysis using the UCSC Xena tool confirmed higher expression of these four genes in breast cancer tissues than in normal tissues. *In vitro* quantification in breast tumor tissues further confirmed the overexpression of these novel identified BRCA biomarkers. The association of these genes with various clinico-pathological parameters in breast cancer patients suggests that these identified genes could be used as potential therapeutic biomarkers in breast cancer patients. Pathway analysis conducted using the biological pathway revealed the involvement of these identified genes in the regulation of key cellular processes, including cell growth, which is critical for cancer development and progression. In addition, analysis of the COSMIC and cBioPortal databases showed that aberrant expression of these novel genes in breast cancer is associated with mutations and genetic alterations. These findings provide valuable insights for researchers investigating the molecular mechanisms of breast cancer. They also provide clinicians with potential targets that could improve diagnostic accuracy and contribute to the development of more effective treatment strategies.

The identification of CACNG*4* as a potential breast cancer biomarker is an important step towards improving clinical outcomes for breast cancer patients. As a transmembrane type I, AMPA receptor regulatory protein, *CACNG4* plays a critical role in regulating both channel gating and trafficking of AMPA receptors [24].

Amplification of *CACNG4* has been shown to contribute to increased breast cancer cell motility, transformation, and metastasis, highlighting the importance of targeted therapies that can disrupt its actions [25]. As CACNG4 is located on the plasma membrane, antibody-based therapies have the potential to inhibit its function and impede breast cancer progression, providing a viable and valuable approach for the development of novel treatment strategies. Additionally, our findings in the biological pathway analysis revealed that *CACNG4* is involved in the ErbB receptor signaling network and the mTOR signaling pathway, both of which have been implicated in cancer metastasis and poor prognosis based on studies by Drago et al. and Tian et al. [26, 27]. It is worth noting that the molecular function of *CACNG4* is voltage-gated calcium channel activity. Studies have shown that calcium channel antagonists have anti-proliferative effects on various cell types, including vascular, retinal pigment, and prostate cancer cells. Therefore, targeting Ca^2+^ pumps or channels has been suggested as a potential therapeutic approach for the treatment of breast cancer [28].

The protein kinase PKMYT1 (Membrane Associated Tyrosine/Threonine 1), a member of the WEE kinase family, has been shown to play a negative role in the G2/M phase of the cell cycle and has been implicated in the development and progression of several cancers, including hepatic, glioblastoma, colorectal, and non-small cell lung cancers [29]. Overexpression of *PKMYT1* in these cancers is typically associated with poor prognosis and disease progression [30]. Based on Kaplan-Meier plotter database analysis, *PKMYT1* overexpression is also associated with a poor prognosis in breast cancer patients. Liu et al. also reported that *PKMYT1* overexpression had been linked to poor prognosis, suggesting that it may be an appealing therapeutic target for breast carcinoma [29]. A study by Zhang et al. demonstrated that *PKMYT1* upregulation promotes tumor progression and correlates with poorer overall survival in patients with esophageal squamous cell carcinoma (ESCC) [31]. In this study, FunRich tool analysis revealed that the biological pathway for co-expressed genes with the *PKMYT1* gene is cell cycle and DNA replication, indicating that overexpression of this gene could develop breast cancer tumorigenesis. This protein upregulation is crucial for the development of some cancers, such as glioblastoma, colon cancer, and hepatic carcinoma [32], and promotes gastric cancer (GC) cell proliferation and apoptosis resistance [33]. This may be due to the effects of *PKMYT1* on enhancing the AKT/mTOR signaling pathway in promoting carcinogenesis and the progression of cancer cells through other pathways, such as activation of Notch signaling [34]. Based on the Cancer Dependency Map analysis tool, lower dependency scores correspond to a higher likelihood that the gene is essential for cell survival or growth. PKMYT1 has been identified as critical for breast cancer cell line survival, suggesting its potential as a viable strategy for therapeutic intervention in breast cancer patients. In another study, *PKMYT1* was identified as a promising target to enhance the radio sensitivity of lung adenocarcinoma (LUAD). This finding suggests that targeting *PKMYT1* could potentially be an attractive target for anticancer therapy [35].

Epiphycan (*EPYC)* is a member of the small leucine-rich repeat proteoglycan family. Epiphycan, also known as dermatan sulfate proteoglycan 3, interacts with collagen fibrils and other extracellular matrix proteins and regulates fibrillogenesis. It has been suggested that *EPYC* is involved in bone formation, maintaining joint integrity, and establishing the organized structure of cartilage through matrix organization [36]. EPYC protein is secreted into the extracellular matrix based on the GenCards database analysis. Studies have shown that insufficient expression of *EPYC* can lead to corneal dystrophy and hearing loss [37]. However, there have been very few studies on the role of *EPYC* in cancer. The FunRich analysis tool revealed that genes co-expressed with *EPYC* are mainly involved in epithelial-mesenchymal transition (EMT), a process in which breast cancer cells acquire mobility, leading to progression and metastasis [38]. A study by Deng et al. investigated the effects of *EPYC* overexpression on the proliferation, invasion, and metastasis of ovarian cancer cells [36]. In the current study, *EPYC* was found to be positively co-expressed with COL11A1 and MMP13. Overexpression of COL11A1 is often associated with an aggressive tumor phenotype and a poor prognosis in many solid tumor types, including pancreatic, breast, ovarian, and colorectal cancers [39]. MMP-13 may be vital for the invasion and metastasis of breast cancer cells [40] and may be helpful as a prognostic marker when assessed simultaneously with lymph node status and HER2 expression [41]. Additionally, Spearman correlation analysis revealed a significant positive correlation between the mRNA expression level of *CACNG4* and *EPYC*, indicating a strong association between the expression of these two genes.

The *CHRNA6* gene encodes an alpha subunit of neuronal nicotinic acetylcholine receptors, which function as ion channels and play a crucial role in neurotransmission in the nervous system. This protein is activated by acetylcholine and exogenous nicotine and mediates dopaminergic neurotransmission. In this study, the CHRNA6 protein is predicted to be expressed on the plasma membrane based on the GenCards database, indicating that antibody-targeted therapy could be helpful. However, there is currently no in silico or experimental study on the effects of the *CHRNA6* gene on cell proliferation and tumor progression, and it could be a potential novel biomarker in cancer studies. This study investigates for the first time the mRNA expression of *CHRNA6* in breast tumor tissues. The FunRich analysis tool revealed that *CHRNA6* interacts with other molecules and is involved in the ErbB receptor signaling network and signal transduction. This pathway plays a crucial role in regulating cell growth and differentiation, and its dysregulation has been implicated in various cancers [27]. The clinico-pathological databases analysis showed that HER2 upregulation and PR downregulation were associated with high *CHRNA6* expression, and BRCA1/2 mutation was associated with low *CHRNA6* expression, suggesting that *CHRNA6* may be a potential diagnostic biomarker in breast cancer. The co-expression of *CHRNA6* with TLR7 and OLR1 was investigated and confirmed. Survival analysis showed that TLR7 expression had a significant impact on survival [42]. OLR1 overexpression revealed a poor prognosis in breast cancer and might represent a potential therapeutic target for breast cancer patients [43].

This retrospective study has some limitations. First, although new breast cancer-associated biomarkers are predicted, their mechanism of action remains unclear. Second, the results need to be validated by a larger sample size and more experimental studies. Therefore, additional prospective clinical and large-scale studies are needed to validate these results.

## Conclusion

The integration of bioinformatics databases could help to find and select novel diagnostic, prognostic, and therapeutic biomarkers. Through bioinformatics analysis and qRT-PCR validation, we confirmed the upregulation of *CACNG4*, *PKMYT1*, *EPYC*, and *CHRNA6* in breast cancer. The co-expression and GO enrichment analyses shed light on the potential mechanisms of these genes in breast cancer development and progression. We propose that *CACNG4*, *PKMYT1*, and *CHRNA6* hold promise as potential targets for both the diagnosis and treatment of breast cancer, while *EPYC* has the potential to be used only as an effective diagnostic biomarker.

### Supplementary Information

**Additional file 1.** List of the differentially expressed genes (DEGs) and the Gini index of the random forest model for each of DEGs.

**Additional file 2: Supplementary Fig. 1A.** Volcano plot of tumor vs. healthy samples. The red points represent the 500 upregulated genes.

**Additional file 3: Supplementary Table 1.** Altered expression of selected genes based on METABRIC database.

**Additional file 4: Supplementary Fig. 2A.** Expression levels of *CACNG4*, *PKMYT1*,*EPYC* and *CHRNA6* in different breast cancer sample types based on the UCSC Xena server from TCGA dataset. High expression levels of identified genes in primary tumor (Blue) compared to normal tissues (Green).

**Additional file 5: Supplementary Fig. 3.** Subcellular Localization Prediction. The GeneCards database was used to evaluate each protein’s subcellular location.

**Additional file 6: Supplementary Table 2.** The correlation between *CACNG4*, *PKMYT1*,*EPYC* and *CHRNA6 *expression levels and clinico-pathological parameters.

**Additional file 7: Supplementary Fig. 4A.** The expression analysis of *CACNG4*, *PKMYT1*,*EPYC* and *CHRNA6* with clinical characteristics of BC patients. A: SBR; B: BRCA1/2 status; C: PAM50 subtypes via bc-GenExMiner v4.8. These graphs were generated by comparing significant changes between normal variables and other variables. Abbreviation: BC, breast cancer; SBR, Scarff Bloom and Richardson grade status.

**Additional file 8.** Gene ontology and biological pathway of co-expressed genes with CACNG4.

**Additional file 9.** Gene ontology and biological pathway of co-expressed genes with PKMYT1.

**Additional file 10.** Gene ontology and biological pathway of co-expressed genes with EPYC.

**Additional file 11.** Gene ontology and biological pathway of co-expressed genes with CHRNA6.

**Additional file 12: Supplementary Fig. 5A.** Venn diagram represents the intersection of genes between the cBioPortal database and the GEPIA2 database. 56 co-expressed genes for *CACNG4*, 49 co-expressed genes for *PKMYT1*, 129 co-expressed genes for EPYC and 150 co-expressed genes for *CHRNA6* based on the FunRich analysis tool.

**Additional file 13:** **Supplementary Table 3.** GSEA of hallmark gene sets. The results of the GSEA showed enrichment in 5 gene sets.

**Additional file 14:** **Supplementary Fig. 6.** Assessment of alteration frequency and mutation types. A. Histogram of the frequency of alterations in queried genes. Queried genes are altered in 404 (37%) of queried patients/samples. The frequency of genetic alteration in *CACNG4*, *PKMYT1*,*EPYC* for breast cancer is mRNA high than other copy number variation, however for *CHRNA6* gene, amplification is the most frequent copy number alteration by searching the cBio Cancer Genomics Portal database. B. An overview of the types of mutation observed. Pie charts demonstrating the mutation types of *CACNG4*,*PKMYT1*, *EPYC* and *CHRNA6* in BC based on results from the COSMIC database. BC, breast cancer.

**Additional file 15:** **Supplementary Fig. 7.** Tumor cell line dependency based on the DepMap tool. A: The essential role of indicated genes (*CACNG4*, *PKMYT1*,*EPYC* and *CHRNA6*) in tumor cell line panels via DepMap, was established from CRISPR (blue) and RNAi (violet) databases. B: The Chronos dependence scores in breast cancer cells. A higher likelihood that the gene of interest is crucial in a particular cell line is indicated by a lower Chronos score. A gene is not essential if it has a score of 0 (dotted line); a value of 1 is like the average of all pan-essential genes (red line).

**Additional file 16:** **Supplementary Table 4.** Association of gene expression pattern of *CACNG4*,*PKMYT1*, *EPYC*, and *CHRNA6* with clinico-pathological features in breast cancer patients. This file shows the expression patterns of these genes with age, ER status, PR status, HER2 status, TNM stages, histological grades and molecular subtypes. *P* value less than 0.05 considered to be statistically significant. SD: Significantly difference; NS: Non Significant; ER: Estrogen Receptor; PR: Progesterone Receptor; HER2: Human Epidermal Growth Factor Receptor 2; and TNM: Tumor, Node, Metastasis.

## Data Availability

The data can be available from the corresponding author upon reasonable request. The datasets analysed during the current study are publicly available at: 1. The raw data were obtained from the TCGA database (https://portal.gdc.cancer.gov/) using the TCGAbiolinks R package. 2. The online database GEPIA2 (http://gepia2.cancer-pku.cn/#index). 3. The UCSC Xena (http://xena.ucsc.edu) platform. 4. The UALCAN web resource (https://ualcan.path.uab.edu/). 5. GeneCards database (https://www.genecards.org/). 6. Breast Cancer Gene-Expression Miner v4.8 analysis (bc-GenExMiner v4.8). 7. The cBioPortal database (https://www.cbioportal.org/). 8. The COSMIC database (cancer.sanger.ac.uk). 9. Kaplan-Meier (KM) plotter database (https://kmplot.com/analysis/). 10. The Cancer Dependency Map (https://depmap.org/portal/).

## Abbreviations

BC: Breast Cancer
BRCA: Breast Invasive Carcinoma
ER: Estrogen Receptor
PR: Progesterone Receptor
TNBC: Triple-Negative Breast Cancer
SBR: The Scarff–Bloom–Richardson grade
OS: Overall Survival
DEGs: Differentially Expressed Genes
GO: Gene Ontology
TCGA: The Cancer Genome Atlas
BP: Biological Process
CC: Cellular Component
MF: Molecular Function
GSEA: Gene Set Enrichment Analysis
