# Supplementary material for: Unveiling promising breast cancer biomarkers: an integrative approach combining bioinformatics analysis and experimental verification
Source: BMC Cancer. 2024 Jan 31;24:155. doi: 10.1186/s12885-024-11913-7 (PMC10829368; doi:10.1186/s12885-024-11913-7)
Supplement: Supplementary file 2 — Additional file 2: Supplementary Fig. 1A. Volcano plot of tumor vs. healthy samples. The red points represent the 500 upregulated genes. [file 12885_2024_11913_MOESM2_ESM.doc]

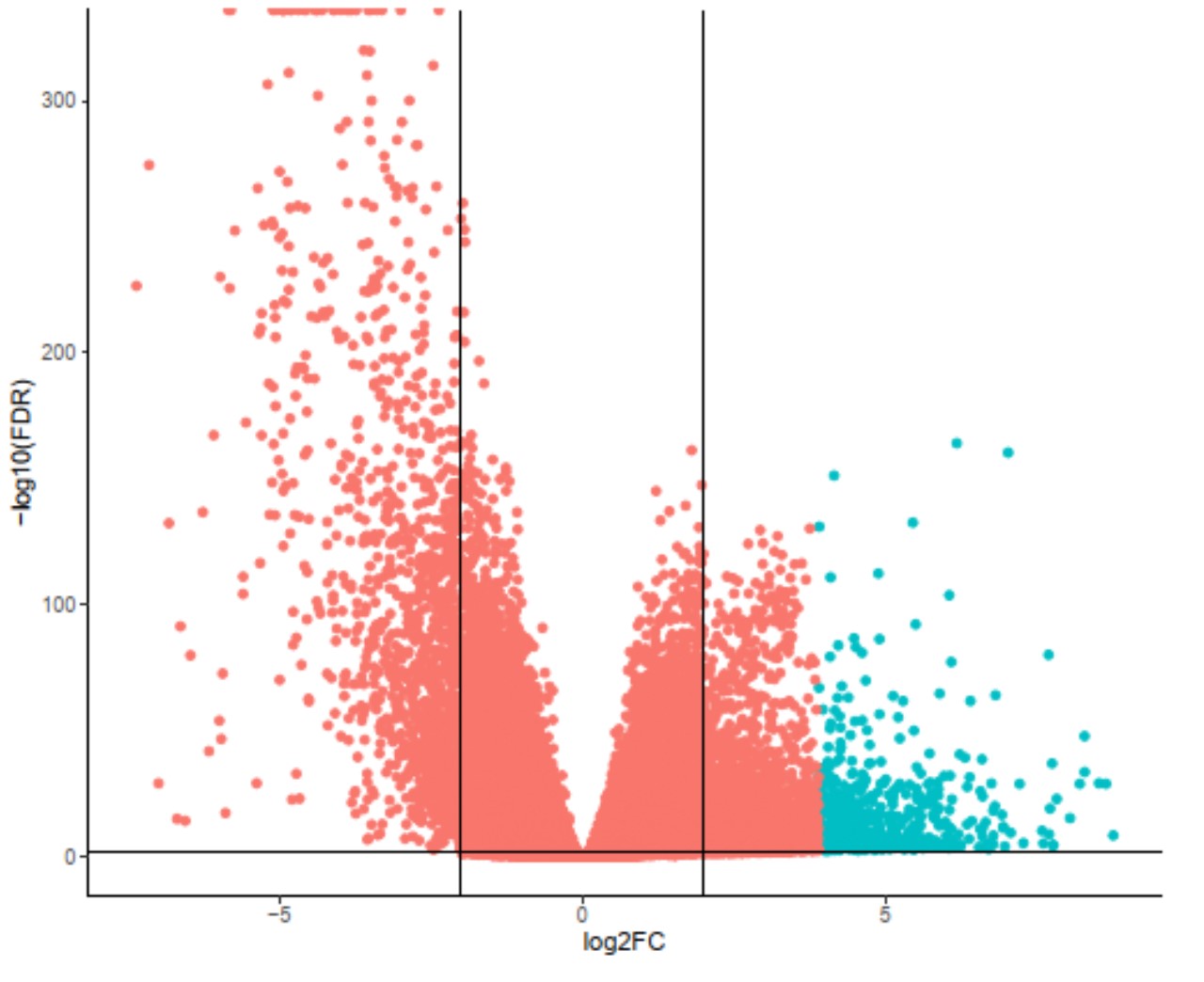


**Supplementary Fig.1A**: Volcano plot of tumor vs. healthy samples. The red points represent the 500 upregulated genes.


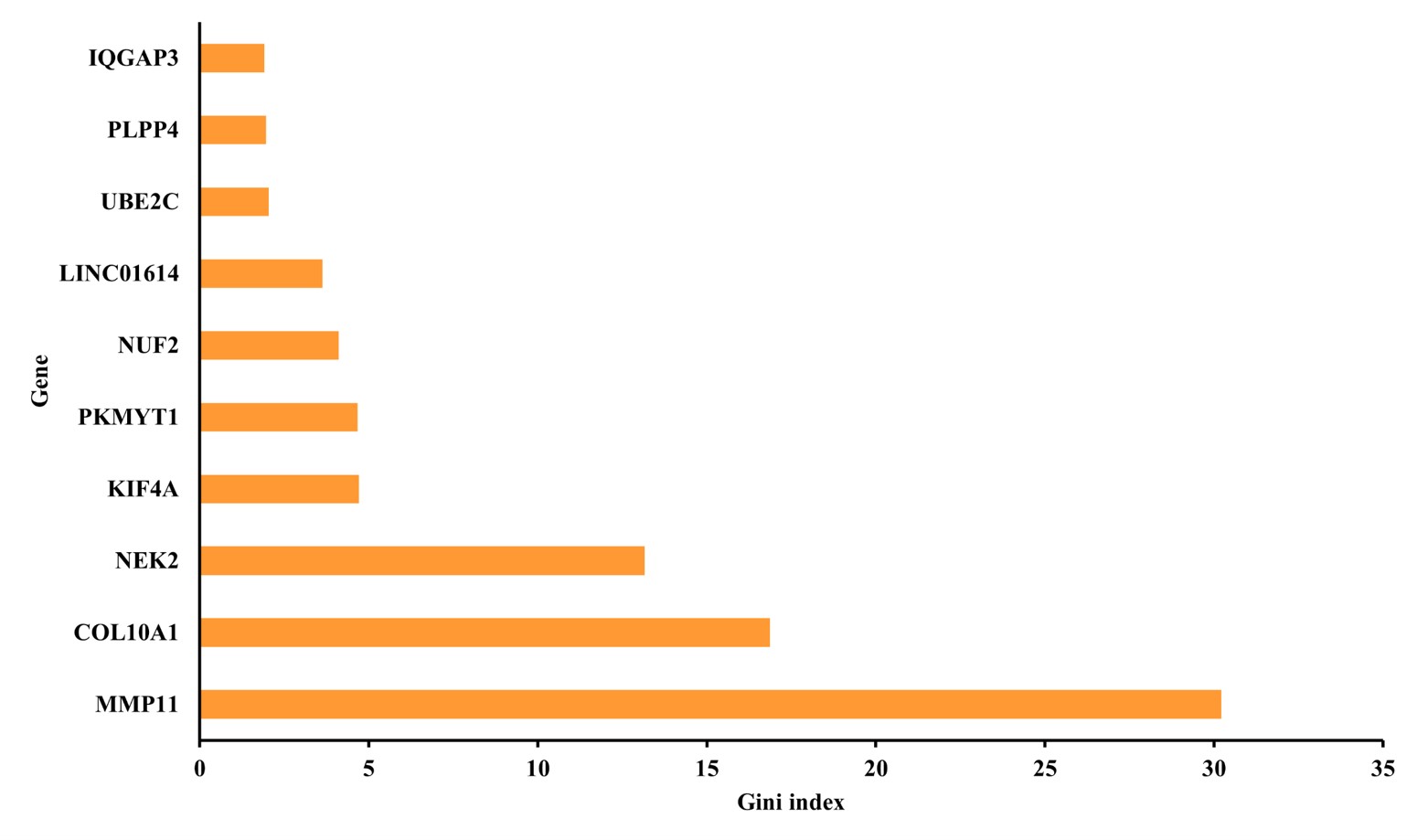


**Supplementary Fig.1B**: Random forest-based assessment of feature importance: visualizing the top 10 variables by Gini index.
