# Supplementary material for: Unveiling promising breast cancer biomarkers: an integrative approach combining bioinformatics analysis and experimental verification
Source: BMC Cancer. 2024 Jan 31;24:155. doi: 10.1186/s12885-024-11913-7 (PMC10829368; doi:10.1186/s12885-024-11913-7)
Supplement: Supplementary file 3 — Additional file 3: Supplementary Table 1. Altered expression of selected genes based on METABRIC database. [file 12885_2024_11913_MOESM3_ESM.doc]

| **Genes** | **logFC** | **FDR** |
| --- | --- | --- |
| PKMYT1 | 2.94 | 1.58E-08 |
| CACNG4 | 3.51 | 2.77E-07 |
| CHRNA6 | 2.14 | 0.0336 |
| EPYC | 1.34 | 0.073 |

**Supplementary Table 1:** Altered expression of selected genes based on METABRIC database.
