# Supplementary material for: Unveiling promising breast cancer biomarkers: an integrative approach combining bioinformatics analysis and experimental verification
Source: BMC Cancer. 2024 Jan 31;24:155. doi: 10.1186/s12885-024-11913-7 (PMC10829368; doi:10.1186/s12885-024-11913-7)
Supplement: Supplementary file 4 — Additional file 4: Supplementary Fig. 2A. Expression levels of CACNG4, PKMYT1,EPYC and CHRNA6 in different breast cancer sample types based on the UCSC Xena server from TCGA dataset. High expression levels of identified genes in primary tumor (Blue) compared to normal tissues (Green). [file 12885_2024_11913_MOESM4_ESM.doc]

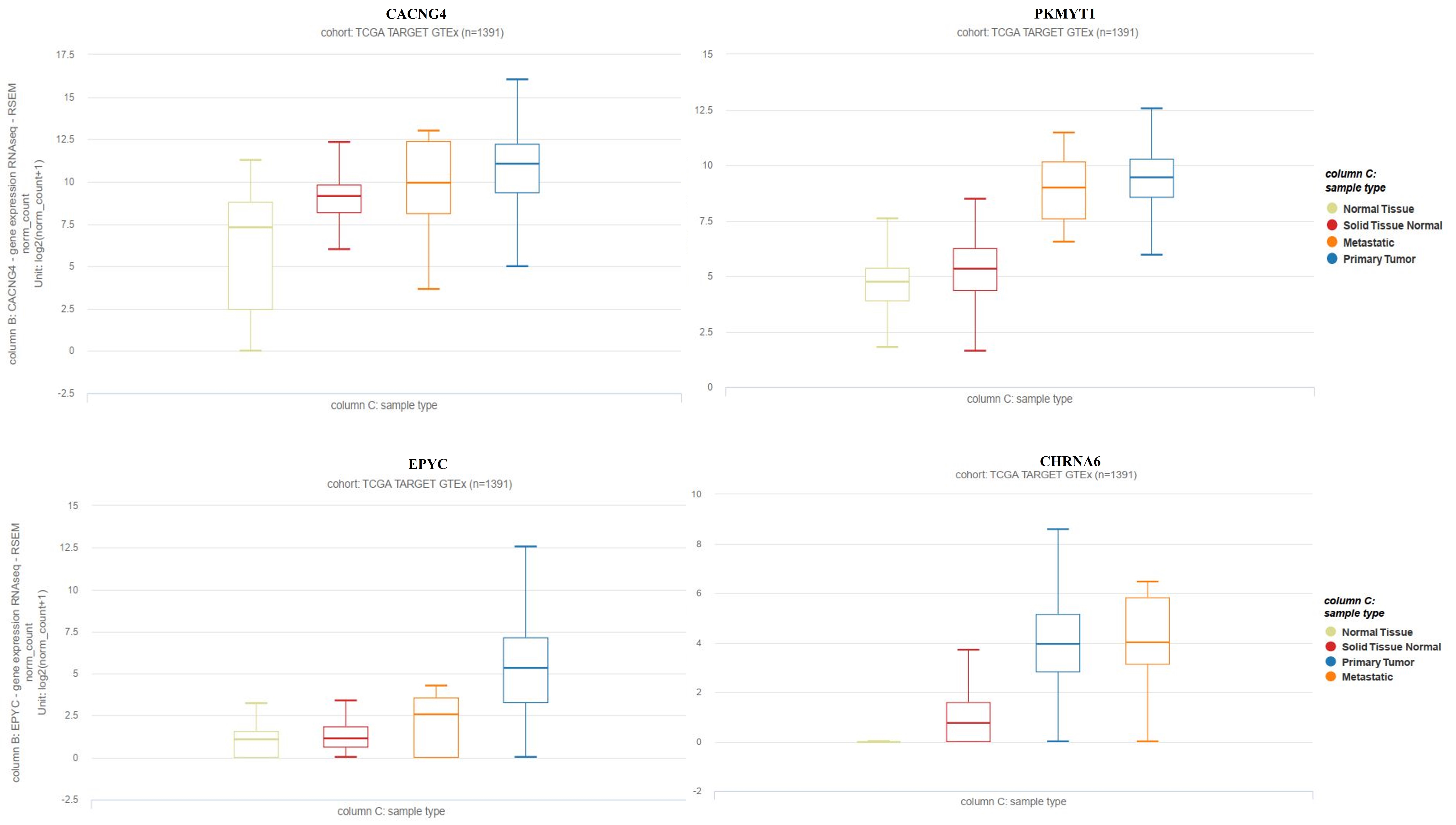


**Supplementary Fig.2A**: Expression levels of *CACNG4*, *PKMYT1*, *EPYC* and *CHRNA6* in different breast cancer sample types based on the UCSC Xena server from TCGA dataset. High expression levels of identified genes in primary tumor (Blue) compared to normal tissues (Green).


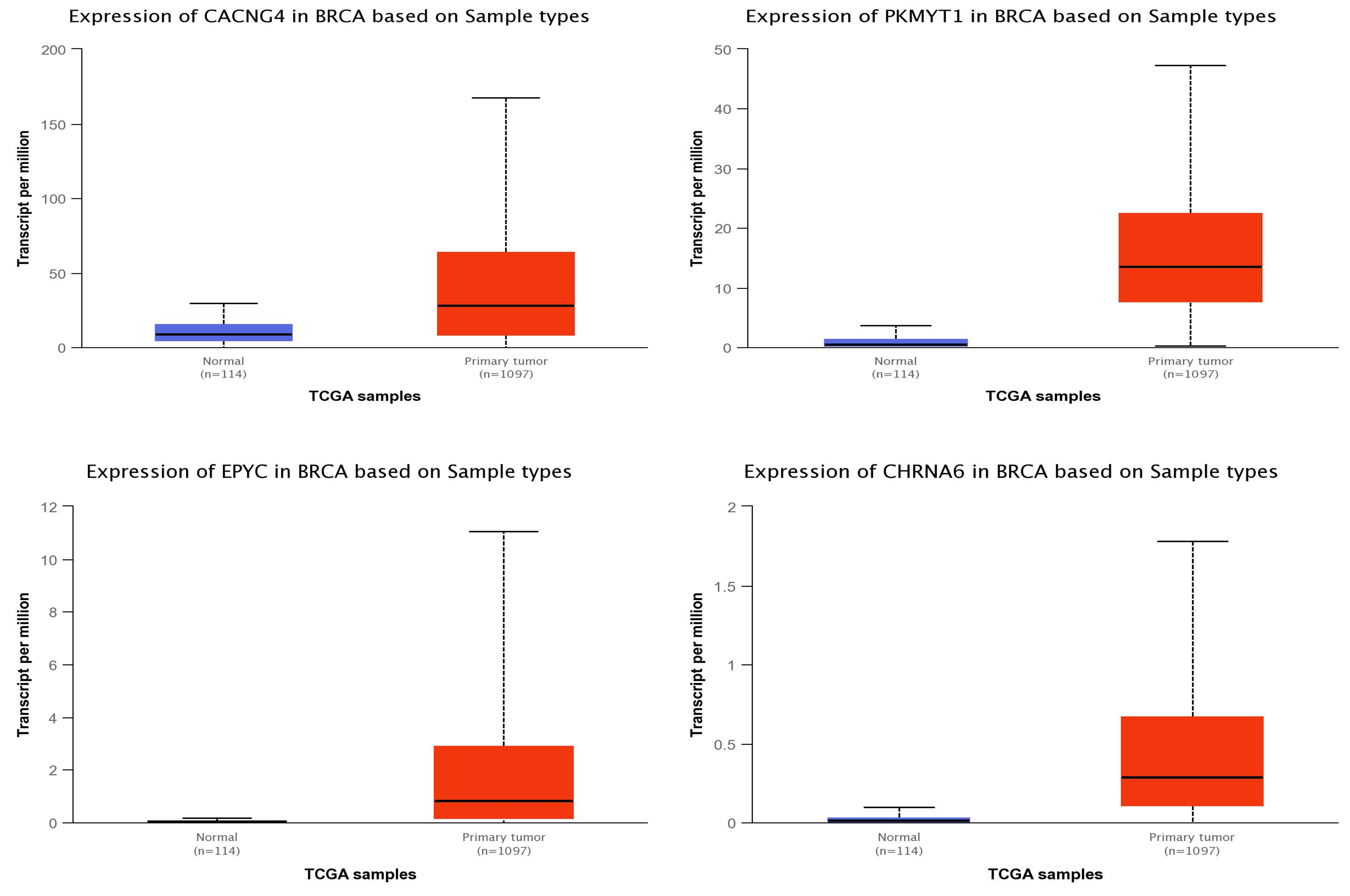


**Supplementary Fig.2B**: Expression levels based on UALCAN database. Expression of *CACNG4*, *PKMYT1*, *EPYC* and *CHRNA6* in BRCA via sample types (primary tumor vs normal). BRCA: Breast Invasive Carcinoma
