# Supplementary material for: Unveiling promising breast cancer biomarkers: an integrative approach combining bioinformatics analysis and experimental verification
Source: BMC Cancer. 2024 Jan 31;24:155. doi: 10.1186/s12885-024-11913-7 (PMC10829368; doi:10.1186/s12885-024-11913-7)
Supplement: Supplementary file 5 — Additional file 5: Supplementary Fig. 3. Subcellular Localization Prediction. The GeneCards database was used to evaluate each protein’s subcellular location. [file 12885_2024_11913_MOESM5_ESM.doc]

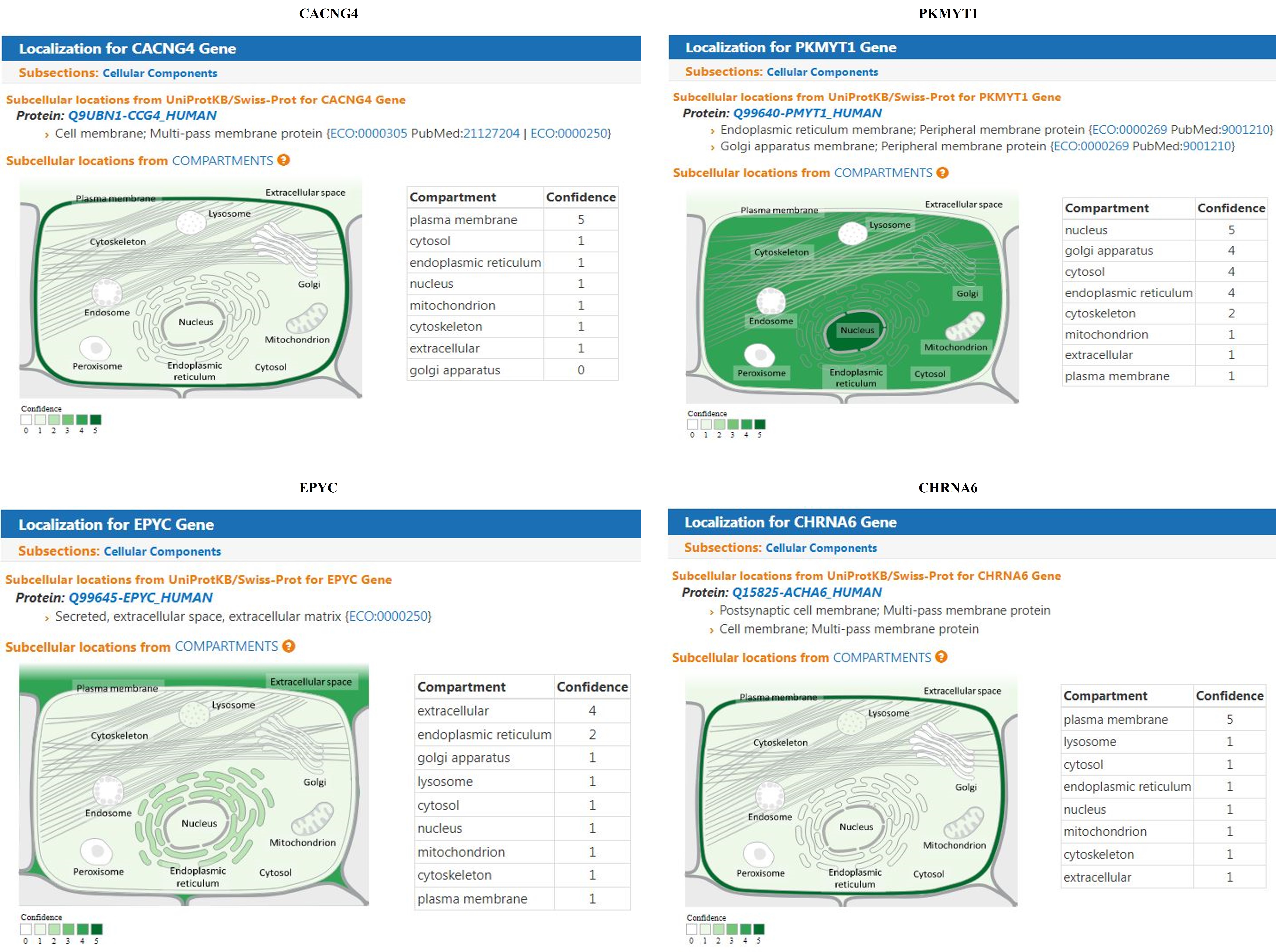


**Supplementary Fig.3:** **Subcellular Localization Prediction.** The GeneCards database was used to evaluate each protein’s subcellular location.
