# Supplementary material for: Unveiling promising breast cancer biomarkers: an integrative approach combining bioinformatics analysis and experimental verification
Source: BMC Cancer. 2024 Jan 31;24:155. doi: 10.1186/s12885-024-11913-7 (PMC10829368; doi:10.1186/s12885-024-11913-7)
Supplement: Supplementary file 6 — Additional file 6: Supplementary Table 2. The correlation between CACNG4, PKMYT1,EPYC and CHRNA6 expression levels and clinico-pathological parameters. [file 12885_2024_11913_MOESM6_ESM.doc]

**Supplementary table 2: The correlation between *CACNG4*, *PKMYT1*, *EPYC* and *CHRNA6* expression levels and clinico-pathological parameters.**

**The correlation between *CACNG4* expression levels and clinico-pathological parameters**

|  | **Gene Expression Comparisons**  ***CACNG4*** | **P-value** |
| --- | --- | --- |
| **Scarff Bloom & Richardson grade status (SBR)** | SBR3 < SBR2  SBR3 < SBR1  SBR2 = SBR1 | **< 0.0001**  **< 0.01**  >0.10 |
| **BRCA1/2 status** | Wild type = Mutated | P=0.4221 |
| **PAM50 subtypes** | HER2-E > Basal-like  Luminal A > Basal-like  Luminal B > Basal-like  Luminal B > HER2-E  Luminal B > Luminal A  Normal breast-like > Basal-like  Normal breast-like < HER2-E  Normal breast-like < Luminal A  Normal breast-like < Luminal B  Luminal A = HER2-E | **< 0.0001**  **< 0.0001**  **< 0.0001**  **< 0.0001**  **< 0.0001**  **< 0.0001**  **< 0.0001**  **< 0.0001**  **< 0.0001**  >0.10 |
| **Nodal Metastasis status** | Normal-vs-N0  Normal-vs-N1  Normal-vs-N2  Normal-vs-N3  N0-vs-N1  N0-vs-N2  N0-vs-N3  N1-vs-N2  N1-vs-N3  N2-vs-N3 | **<1E-12**  **<1E-12**  **3.78930000044519E-07**  **8.76220000001204E-07**  **2.044000E-04**  **2.952400E-02**  7.155400E-01  5.042800E-01  6.455900E-02  8.478200E-02 |
| **TP53 mutation status** | Normal-vs-TP53-Mutant  Normal-vs-TP53-NonMutant  TP53-Mutant-vs-TP53-NonMutant | **1.49479999489444E-08**  **1.62447832963153E-12**  **4.52850000054461E-07** |
| **Patient’s gender** | Normal-Vs-Male  Normal-Vs-Female  Male- Vs -Female | **7.630600E-04**  **1.62436730732907E-12**  **6.164000E-03** |

**The correlation between *PKMYT1* expression levels and clinico-pathological parameters**

|  | **Gene Expression Comparisons**  ***PKMYT1*** | **P-value** |
| --- | --- | --- |
| **Scarff Bloom & Richardson grade status (SBR)** | SBR2 > SBR1  SBR3 > SBR1  SBR3 > SBR2 | **< 0.0001**  **< 0.0001**  **< 0.0001** |
| **BRCA1/2 status** | Wild type < Mutated | **< 0.0001** |
| **PAM50 subtypes** | Luminal A < Basal-like  Luminal A < HER2-E  Luminal B > Luminal A  Normal breast-like < Basal-like  Normal breast-like < HER2-E  Normal breast-like < Luminal B  Luminal B < HER2-E  Luminal B < Basal-like  HER2-E = Basal-like  Normal breast-like =Luminal A | **< 0.0001**  **< 0.0001**  **< 0.0001**  **< 0.0001**  **< 0.0001**  **< 0.0001**  **< 0.001**  **< 0.01**  >0.10  >0.10 |
| **Nodal Metastasis status** | Normal-vs-N0  Normal-vs-N1  Normal-vs-N2  Normal-vs-N3  N0-vs-N1  N0-vs-N2  N0-vs-N3  N1-vs-N2  N1-vs-N3  N2-vs-N3 | **1.6242562850266E-12**  **<1E-12**  **<1E-12**  **1.62447832963153E-12**  1.572390E-01  2.241400E-01  4.671800E-01  8.423400E-01  8.982300E-02  1.301990E-01 |
| **Patient’s gender** | Normal-Vs-Male  Normal-Vs-Female  Male- Vs -Female | **3.919400E-03**  **1.62436730732907E-12**  1.475400E-01 |
| **TP53 mutation status** | Normal-vs-TP53-Mutant  Normal-vs-TP53-NonMutant  TP53-Mutant-vs-TP53-NonMutant | **<1E-12**  **1.62447832963153E-12**  **1.63913327355658E-12** |

**The correlation between *EPYC* expression levels and clinico-pathological parameters**

|  | **Gene Expression Comparisons**  ***EPYC*** | **P-value** |
| --- | --- | --- |
| **Scarff Bloom & Richardson grade status (SBR)** | SBR1 = SBR2=SBR3 | P=0.1052 |
| **BRCA1/2 status** | Wild type = Mutated | P=0.2312 |
| **PAM50 subtypes** | HER2-E > Basal-like  Luminal A < HER2-E  Luminal B < HER2-E  Normal breast-like < Basal-like  Normal breast-like < HER2-E  Normal breast-like < Luminal A  Normal breast-like < Luminal B  Luminal A > Basal-like  Luminal B > Basal-like  Luminal B = Luminal A | **< 0.0001**  **< 0.0001**  **< 0.0001**  **< 0.0001**  **< 0.0001**  **< 0.0001**  **< 0.0001**  **< 0.05**  <0.10  >0.10 |
| **Nodal Metastasis status** | Normal-vs-N0  Normal-vs-N1  Normal-vs-N2  Normal-vs-N3  N0-vs-N1  N0-vs-N2  N0-vs-N3  N1-vs-N2  N1-vs-N3  N2-vs-N3 | **<1E-12**  **1.11022302462516E-16**  **8.35850000000526E-05**  **3.8324000000034E-05**  5.852600E-01  2.450600E-01  8.835700E-02  1.690360E-01  2.100400E-01  5.397600E-02 |
| **Patient’s gender** | Normal-Vs-Male  Normal-Vs-Female  Male- Vs -Female | 1.300380E-01  **1.62447832963153E-12**  5.081000E-01 |
| **TP53 mutation status** | Normal-vs-TP53-Mutant  Normal-vs-TP53-NonMutant  TP53-Mutant-vs-TP53-NonMutant | **1.01474384450739E-13**  **1.62447832963153E-12**  4.944800E-01 |

**The correlation between *CHRNA6* expression levels and clinico-pathological parameters**

|  | **Gene Expression Comparisons**  ***CHRNA6*** | **P-value** |
| --- | --- | --- |
| **Scarff Bloom & Richardson grade status (SBR)** | SBR3 < SBR1  SBR3 < SBR2  SBR2 = SBR1 | **< 0.0001**  **< 0.0001**  >0.10 |
| **BRCA1/2 status** | Wild type > Mutated | **P=0.0105** |
| **PAM50 subtypes** | Luminal A > Basal-like  Luminal A > HER2-E  Luminal B > Basal-like  Luminal B < Luminal A  Normal breast-like < Luminal A  Luminal B > HER2-E  Normal breast-like < Luminal B  HER2-E > Basal-like  Normal breast-like > Basal-like  Normal breast-like = HER2-E | **< 0.0001**  **< 0.0001**  **< 0.0001**  **< 0.0001**  **< 0.0001**  **< 0.001**  **< 0.01**  **< 0.01**  **< 0.05**  >0.10 |
| **Nodal Metastasis status** | Normal-vs-N0  Normal-vs-N1  Normal-vs-N2  Normal-vs-N3  N0-vs-N1  N0-vs-N2  N0-vs-N3  N1-vs-N2  N1-vs-N3  N2-vs-N3 | **3.980400E-02**  **1.62436730732907E-12**  **4.31754632046477E-12**  **1.96009997122104E-09**  3.066000E-01  3.910600E-01  2.576800E-01  3.659200E-01  4.940000E-01  1.895450E-01 |
| **Patient’s gender** | Normal-Vs-Male  Normal-Vs-Female  Male- Vs -Female | **4.983700E-02**  **1.457770E-03**  1.469560E-01 |
| **TP53 mutation status** | Normal-vs-TP53-Mutant  Normal-vs-TP53-NonMutant  TP53-Mutant-vs-TP53-NonMutant | **1.62458935193399E-12**  **7.083100E-03**  7.348300E-02 |
