# Supplementary material for: Unveiling promising breast cancer biomarkers: an integrative approach combining bioinformatics analysis and experimental verification
Source: BMC Cancer. 2024 Jan 31;24:155. doi: 10.1186/s12885-024-11913-7 (PMC10829368; doi:10.1186/s12885-024-11913-7)
Supplement: Supplementary file 7 — Additional file 7: Supplementary Fig. 4A. The expression analysis of CACNG4, PKMYT1,EPYC and CHRNA6 with clinical characteristics of BC patients. A: SBR; B: BRCA1/2 status; C: PAM50 subtypes via bc-GenExMiner v4.8. These graphs were generated by comparing significant changes between normal variables and other variables. Abbreviation: BC, breast cancer; SBR, Scarff Bloom and Richardson grade status. [file 12885_2024_11913_MOESM7_ESM.doc]

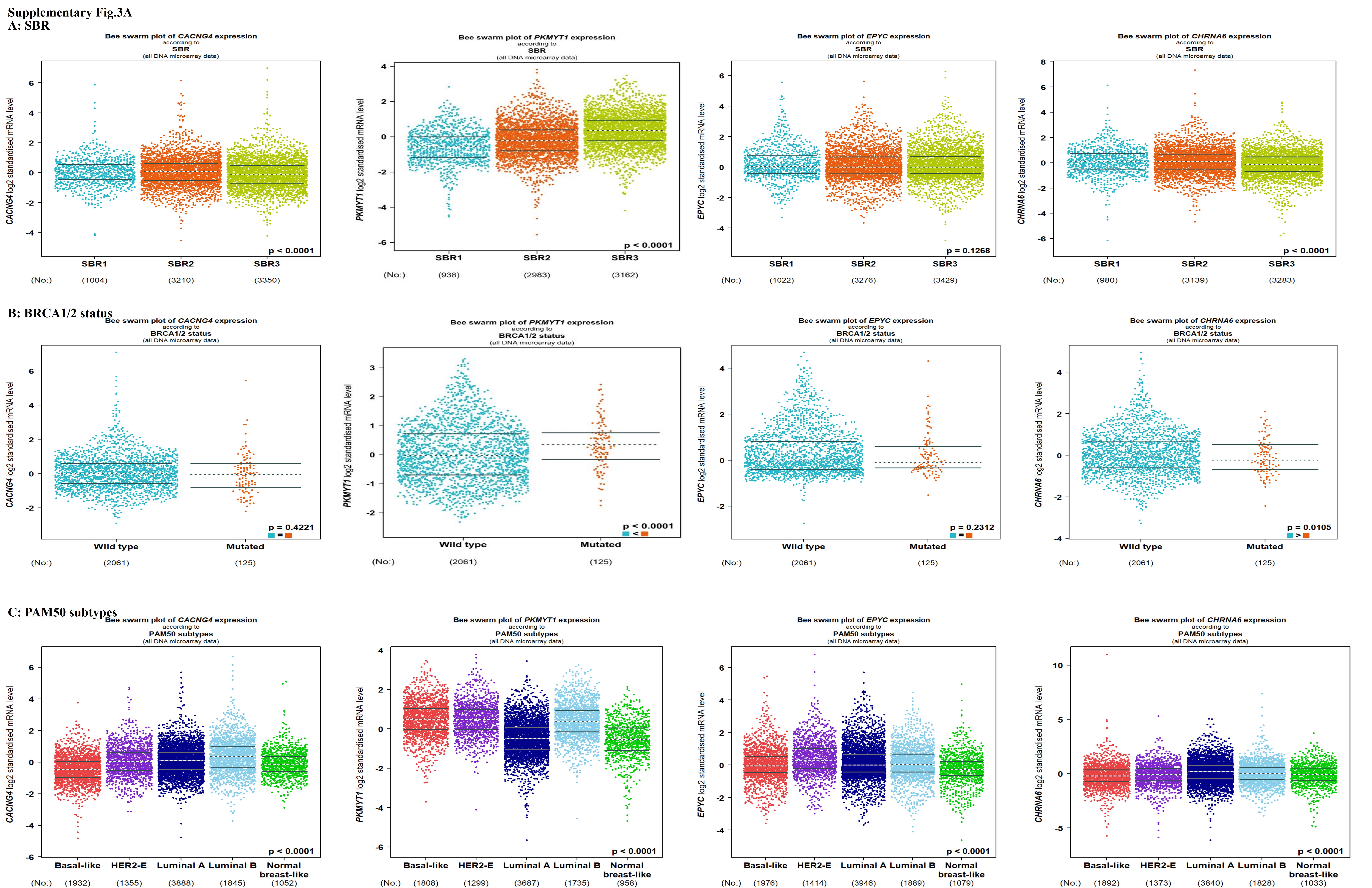


**Supplementary Fig.4A**: The expression analysis of *CACNG4*, *PKMYT1*, *EPYC* and *CHRNA6* with clinical characteristics of BC patients. **A: SBR**; **B: BRCA1/2 status**; **C: PAM50 subtypes** via bc-GenExMiner v4.8. These graphs were generated by comparing significant changes between normal variables and other variables. Abbreviation: BC, breast cancer; SBR, Scarff Bloom and Richardson grade status.


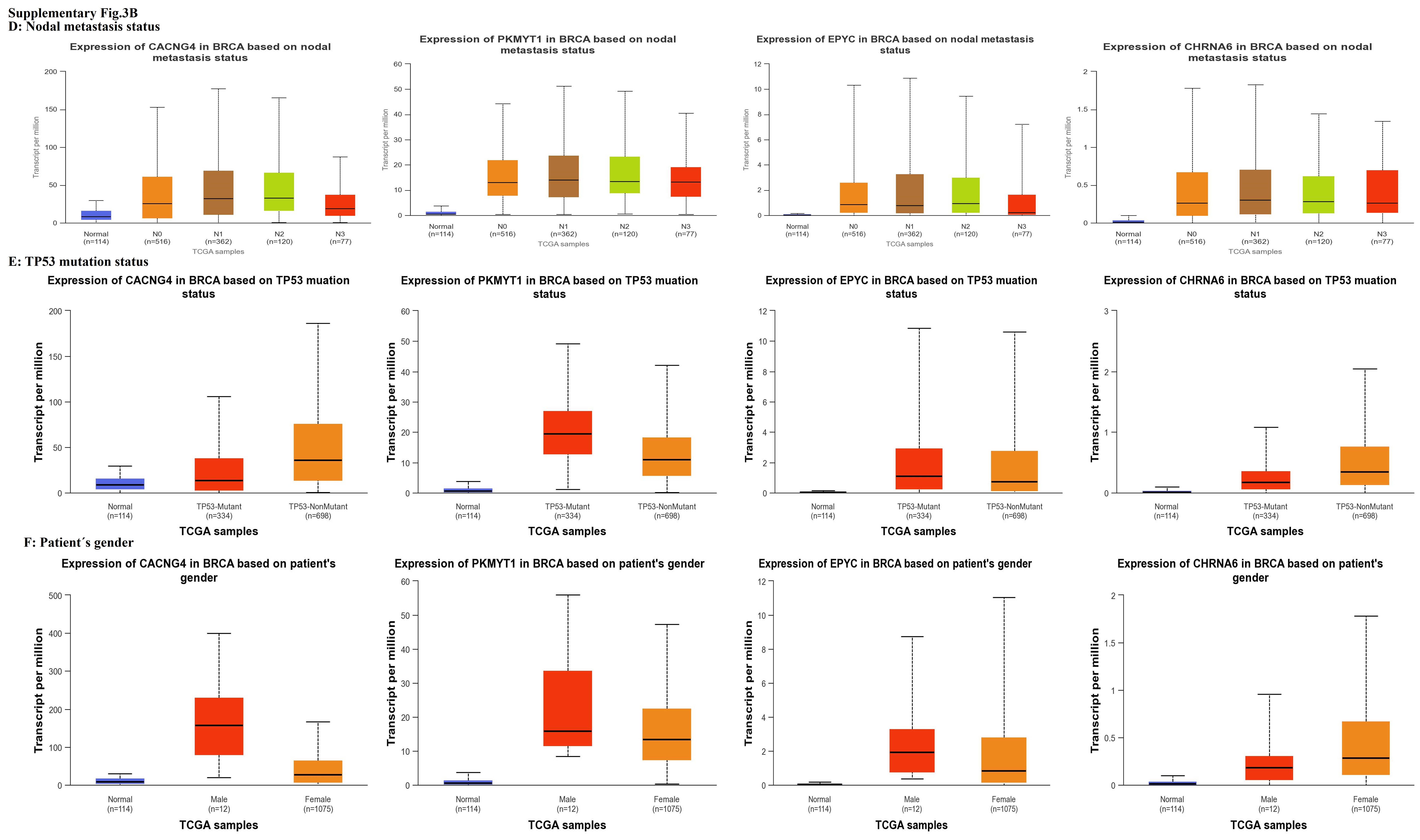


**Supplementary Fig.4B**: The expression analysis of *CACNG4*, *PKMYT1*, *EPYC* and *CHRNA6* with clinical characteristics of BRCA patients. **D: Nodal metastasis status**; **E: TP53 mutation status** and **F: Patient´s gender** through UALCAN database.Abbreviation: BRCA, breast invasive carcinoma; N0, no regional lymph node metastasis; N1, metastases in 1–3 axillary lymph nodes; N2, metastases in 4–9 axillary lymph nodes; N3, metastases in 10 or more axillary lymph nodes.
