# Supplementary material for: Unveiling promising breast cancer biomarkers: an integrative approach combining bioinformatics analysis and experimental verification
Source: BMC Cancer. 2024 Jan 31;24:155. doi: 10.1186/s12885-024-11913-7 (PMC10829368; doi:10.1186/s12885-024-11913-7)
Supplement: Supplementary file 12 — Additional file 12: Supplementary Fig. 5A. Venn diagram represents the intersection of genes between the cBioPortal database and the GEPIA2 database. 56 co-expressed genes for CACNG4, 49 co-expressed genes for PKMYT1, 129 co-expressed genes for EPYC and 150 co-expressed genes for CHRNA6 based on the FunRich analysis tool. [file 12885_2024_11913_MOESM12_ESM.doc]

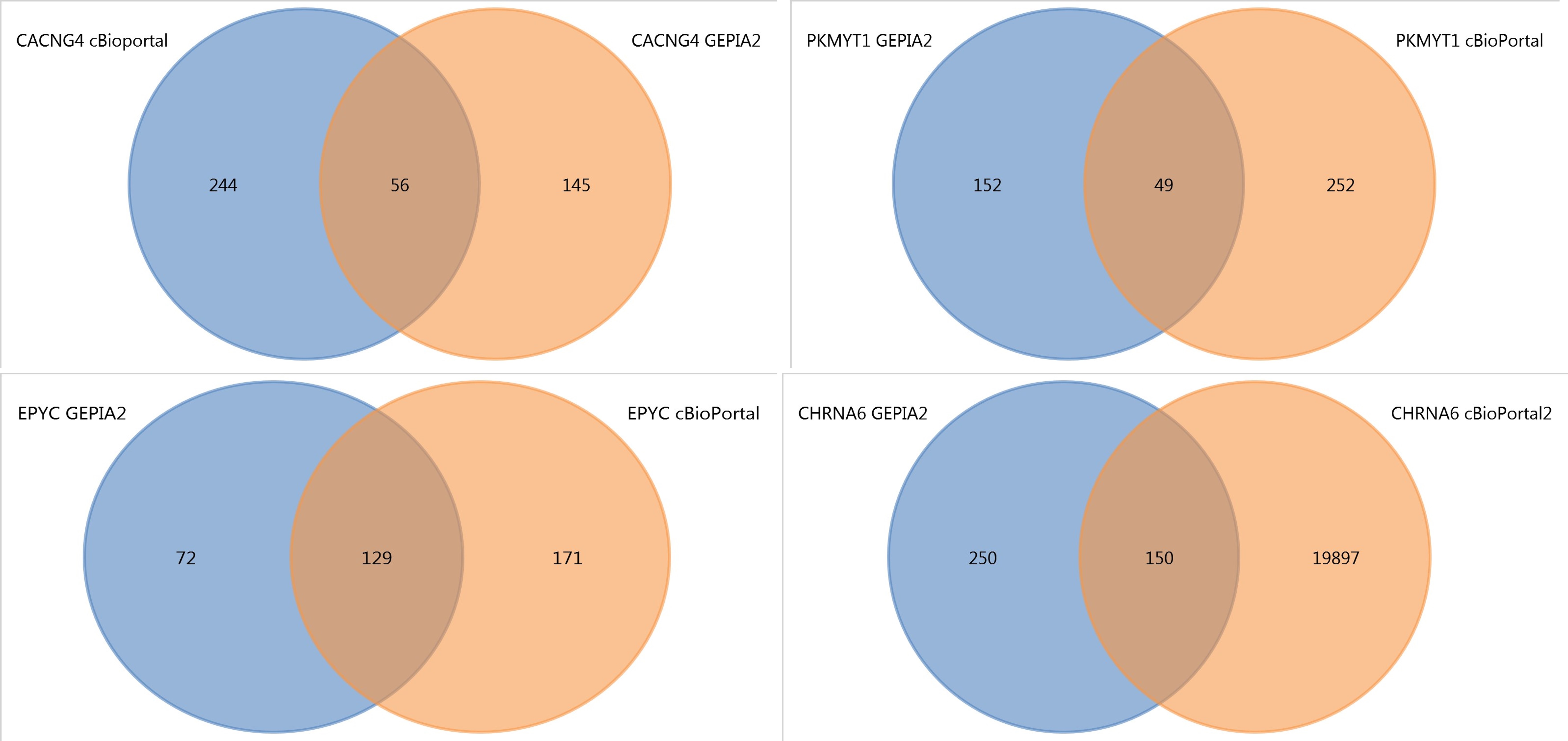


**Supplementary Fig.5A**: Venn diagram represents the intersection of genes between the cBioPortal database and the GEPIA2 database. 56 co-expressed genes for *CACNG4*, 49 co-expressed genes for *PKMYT1*, 129 co-expressed genes for *EPYC* and 150 co-expressed genes for *CHRNA6* based on the FunRich analysis tool.


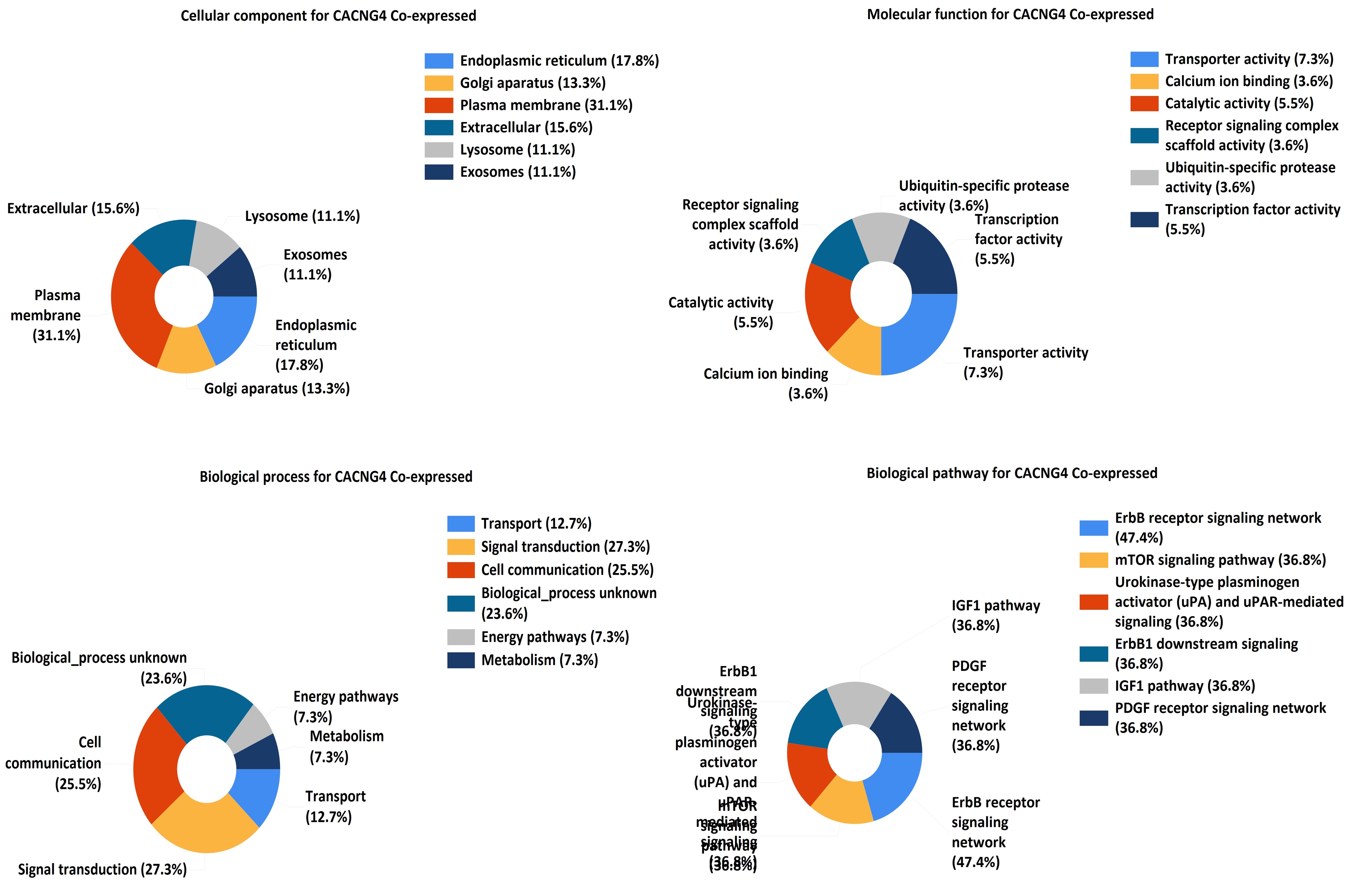


**Supplementary Fig.5B:** KEGG and GO enrichment analyses of co-expressed genes according to the FunRich tool. GO enrichment of co-expressed genes for cellular component, molecular function, biological process and biological pathway in *CACNG4*. KEGG, Kyoto Encyclopedia of Genes and Genomes; GO, Gene Ontology.


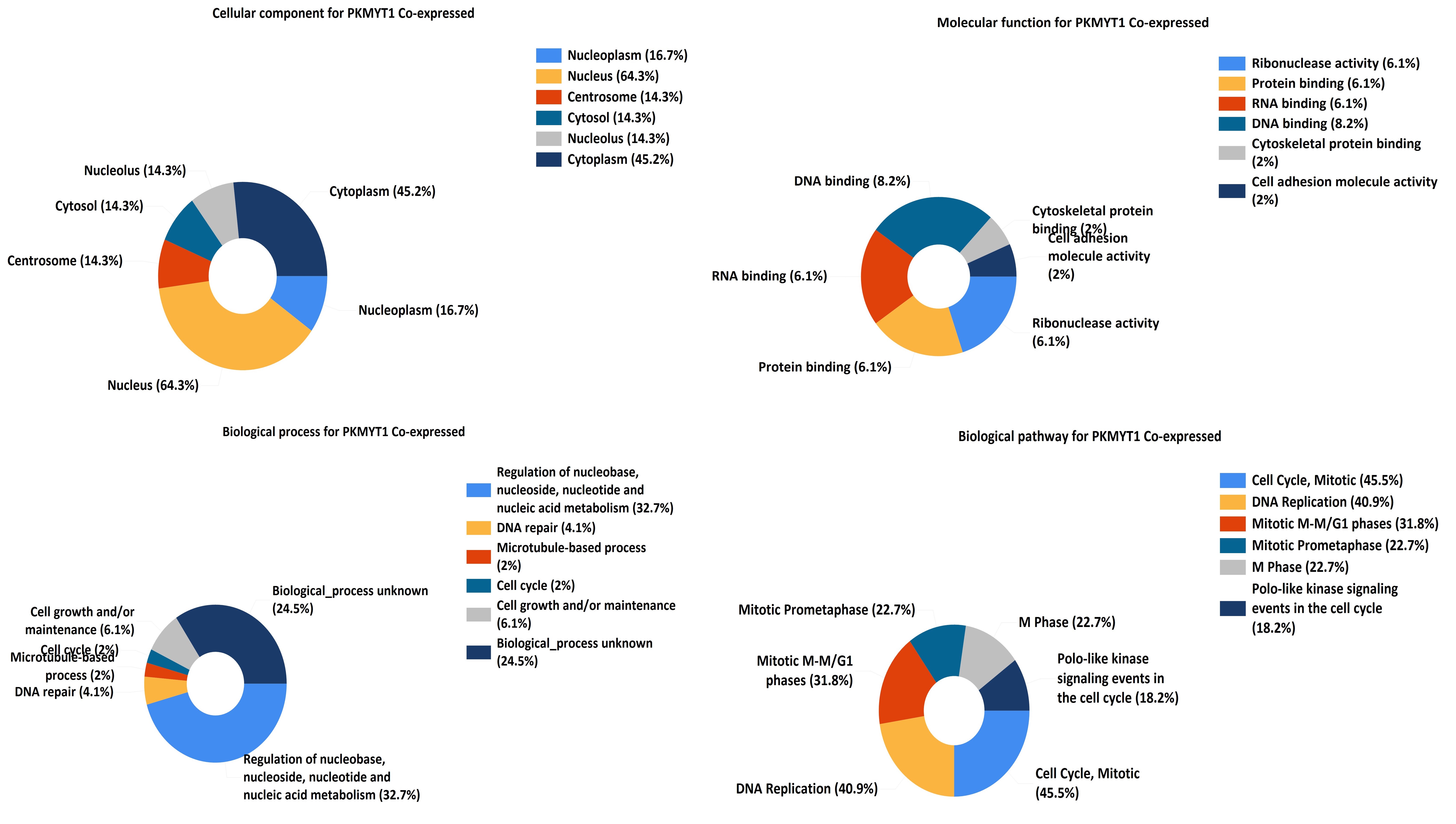


**Supplementary Fig.5C:** KEGG and GO enrichment analyses of co-expressed genes according to the FunRich tool. GO enrichment of co-expressed genes for cellular component, molecular function, biological process and biological pathway in *PKMYT1*. KEGG, Kyoto Encyclopedia of Genes and Genomes; GO, Gene Ontology.


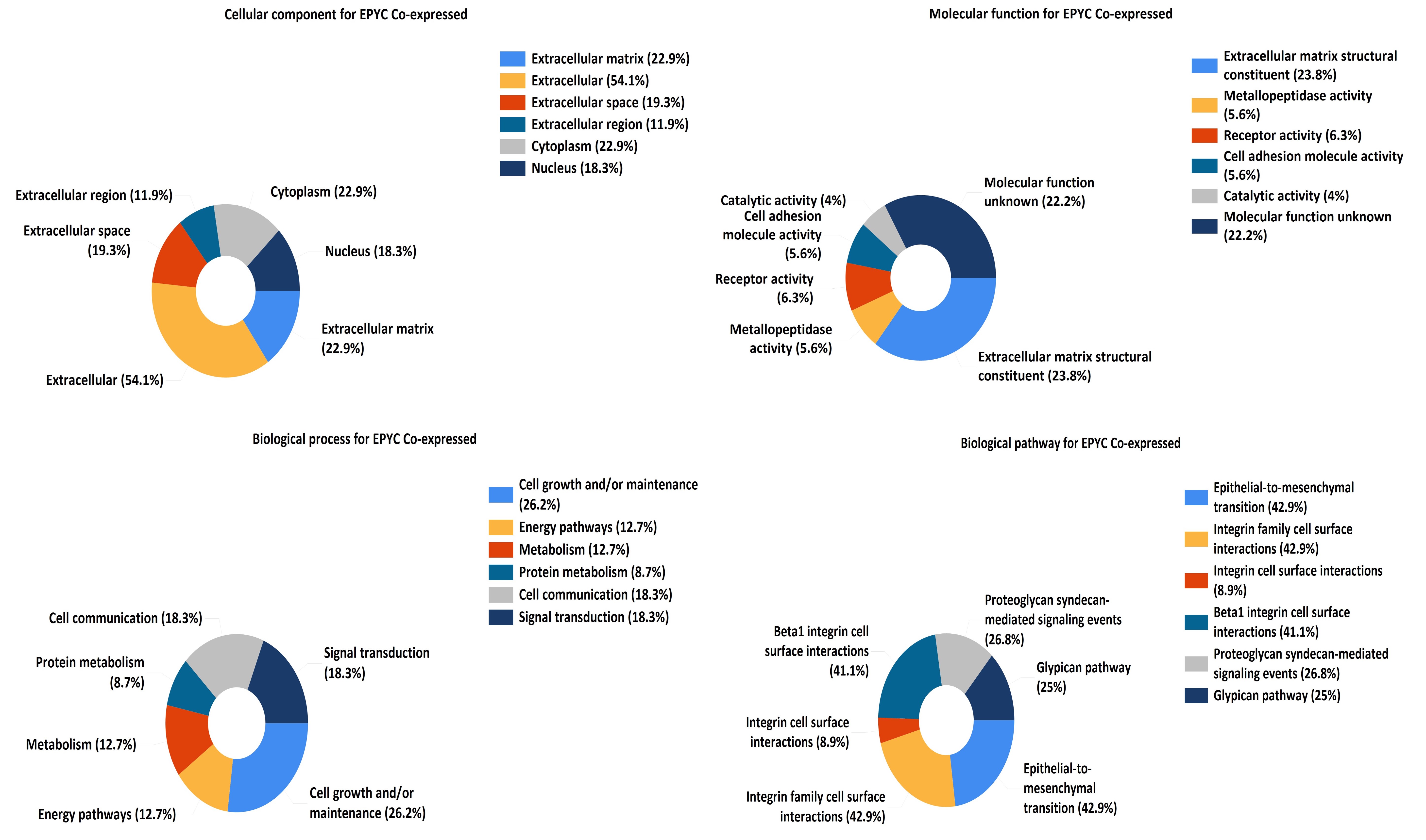


**Supplementary Fig.5D:** KEGG and GO enrichment analyses of co-expressed genes according to the FunRich tool. GO enrichment of co-expressed genes for cellular component, molecular function, biological process and biological pathway in *EPYC*. KEGG, Kyoto Encyclopedia of Genes and Genomes; GO, Gene Ontology.


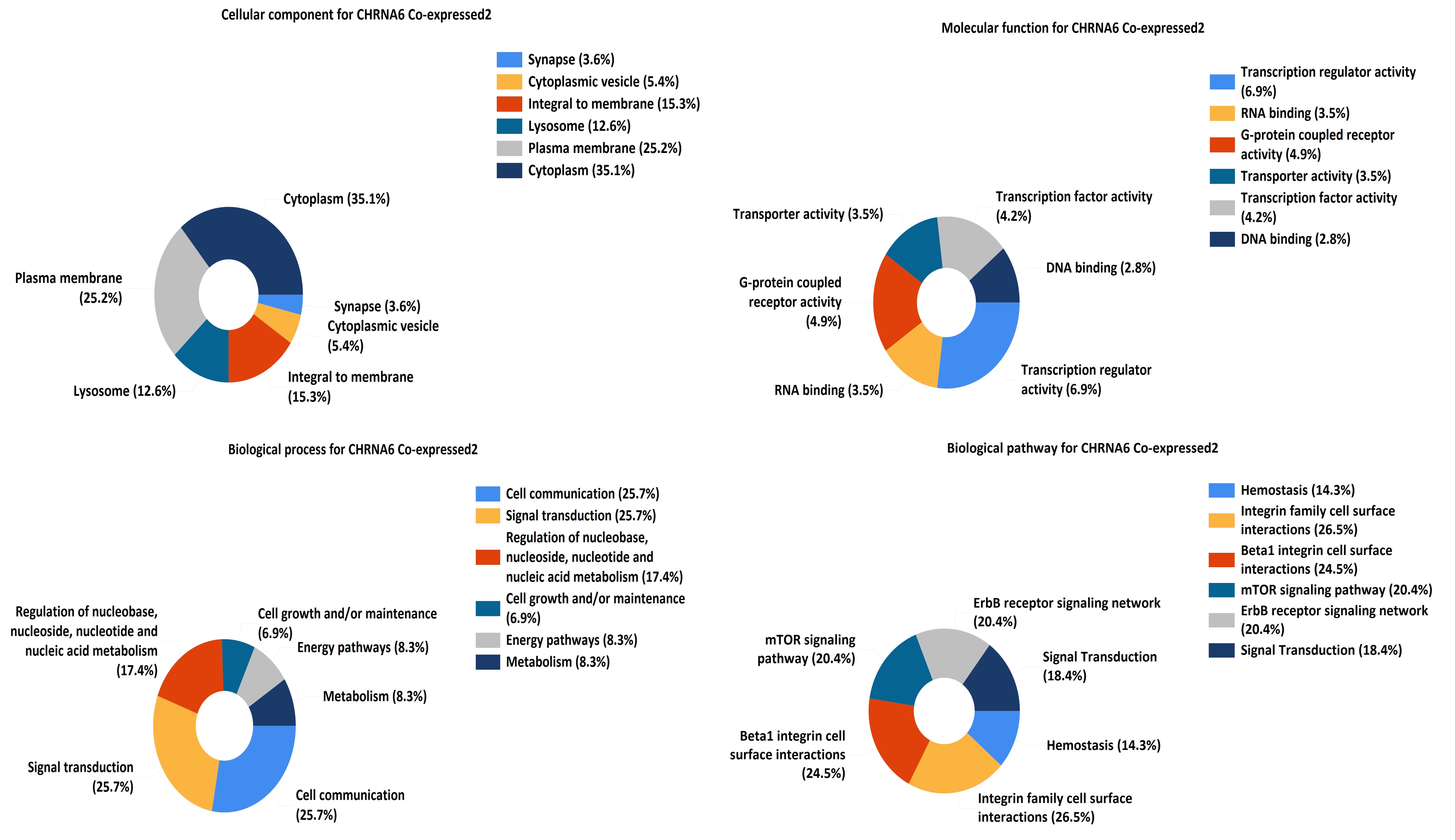


**Supplementary Fig.5E:** KEGG and GO enrichment analyses of co-expressed genes according to the FunRich tool. GO enrichment of co-expressed genes for cellular component, molecular function, biological process and biological pathway in *CHRNA6*. KEGG, Kyoto Encyclopedia of Genes and Genomes; GO, Gene Ontology.
