# Supplementary material for: Unveiling promising breast cancer biomarkers: an integrative approach combining bioinformatics analysis and experimental verification
Source: BMC Cancer. 2024 Jan 31;24:155. doi: 10.1186/s12885-024-11913-7 (PMC10829368; doi:10.1186/s12885-024-11913-7)
Supplement: Supplementary file 13 — Additional file 13: Supplementary Table 3. GSEA of hallmark gene sets. The results of the GSEA showed enrichment in 5 gene sets. [file 12885_2024_11913_MOESM13_ESM.doc]

| Summary of Gene set enrichment analysis results with FDR ≤ 0.001. |  | |
| --- | --- | --- |
| GS DETAILS | SIZE | FDR q-val |
| HALLMARK_G2M_CHECKPOINT | 11 | 0 |
| HALLMARK_E2F_TARGETS | 9 | 0 |
| HALLMARK_ESTROGEN_RESPONSE_LATE | 7 | 0 |
| HALLMARK_ESTROGEN_RESPONSE_EARLY | 6 | 0.034 |
| HALLMARK_EPITHELIAL_MESENCHYMAL_TRANSITION | 33 | 0.0405 |
| HALLMARK_MYOGENESIS | 6 | 0.046 |

**Supplementary Table 3:** GSEA of hallmark gene sets. The results of the GSEA showed enrichment in 5 gene sets.
