# Supplementary material for: Unveiling promising breast cancer biomarkers: an integrative approach combining bioinformatics analysis and experimental verification
Source: BMC Cancer. 2024 Jan 31;24:155. doi: 10.1186/s12885-024-11913-7 (PMC10829368; doi:10.1186/s12885-024-11913-7)
Supplement: Supplementary file 14 — Additional file 14: Supplementary Fig. 6. Assessment of alteration frequency and mutation types. A. Histogram of the frequency of alterations in queried genes. Queried genes are altered in 404 (37%) of queried patients/samples. The frequency of genetic alteration in CACNG4, PKMYT1,EPYC for breast cancer is mRNA high than other copy number variation, however for CHRNA6 gene, amplification is the most frequent copy number alteration by searching the cBio Cancer Genomics Portal database. B. An overview of the types of mutation observed. Pie charts demonstrating the mutation types of CACNG4,PKMYT1, EPYC and CHRNA6 in BC based on results from the COSMIC database. BC, breast cancer. [file 12885_2024_11913_MOESM14_ESM.doc]

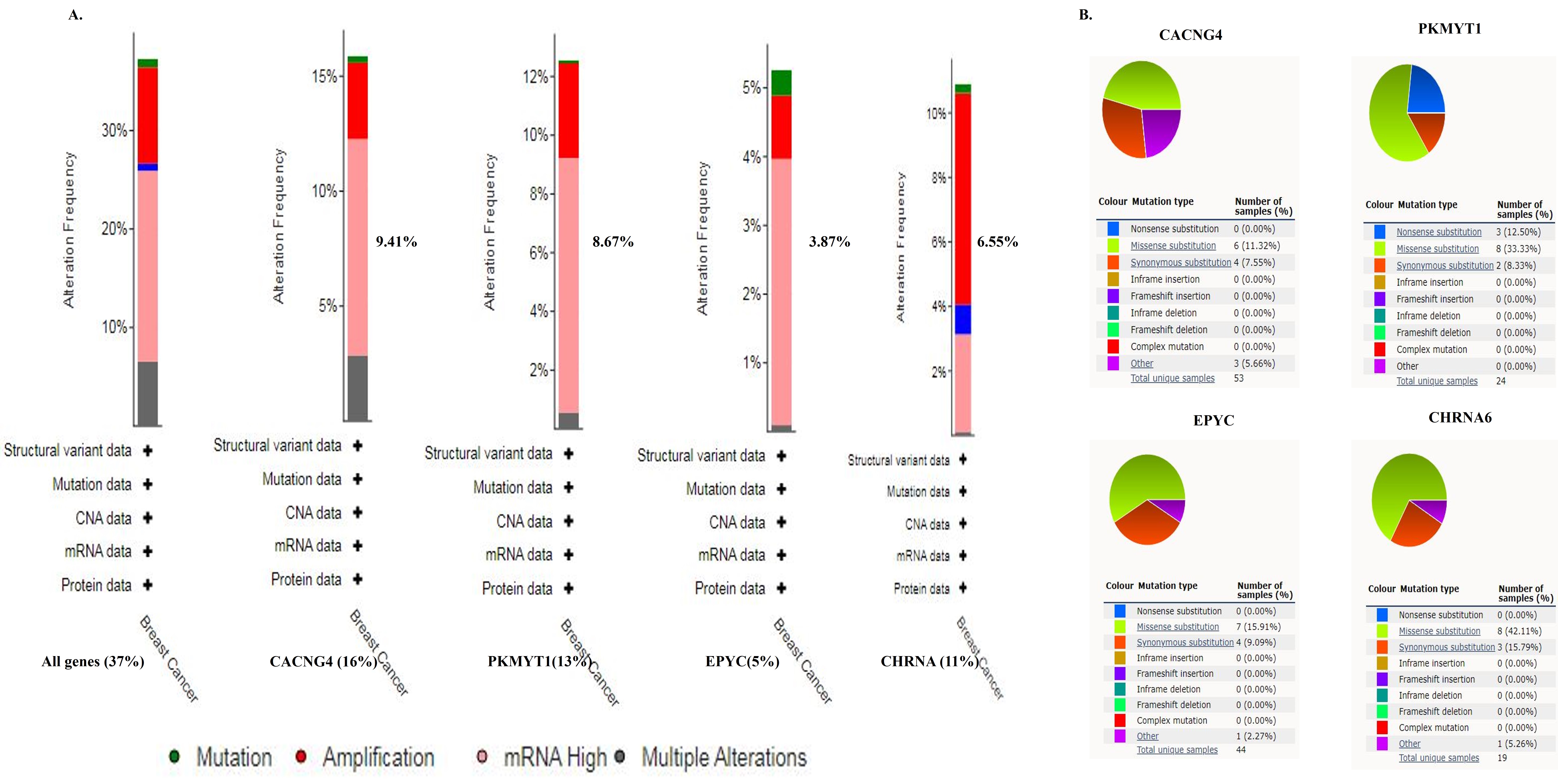


**Supplementary Fig.6**: **Assessment of alteration frequency and mutation types.** **A.** Histogram of the frequency of alterations in queried genes. Queried genes are altered in 404 (37%) of queried patients/samples. The frequency of genetic alteration in *CACNG4*, *PKMYT1*, *EPYC* for breast cancer is mRNA high than other copy number variation, however for *CHRNA6* gene, amplification is the most frequent copy number alteration by searching the cBio Cancer Genomics Portal database. **B.** An overview of the types of mutation observed. Pie charts demonstrating the mutation types of *CACNG4*, *PKMYT1*, *EPYC* and *CHRNA6* in BC based on results from the COSMIC database. BC, breast cancer.
