# Supplementary material for: Unveiling promising breast cancer biomarkers: an integrative approach combining bioinformatics analysis and experimental verification
Source: BMC Cancer. 2024 Jan 31;24:155. doi: 10.1186/s12885-024-11913-7 (PMC10829368; doi:10.1186/s12885-024-11913-7)
Supplement: Supplementary file 15 — Additional file 15: Supplementary Fig. 7. Tumor cell line dependency based on the DepMap tool. A: The essential role of indicated genes (CACNG4, PKMYT1,EPYC and CHRNA6) in tumor cell line panels via DepMap, was established from CRISPR (blue) and RNAi (violet) databases. B: The Chronos dependence scores in breast cancer cells. A higher likelihood that the gene of interest is crucial in a particular cell line is indicated by a lower Chronos score. A gene is not essential if it has a score of 0 (dotted line); a value of 1 is like the average of all pan-essential genes (red line). [file 12885_2024_11913_MOESM15_ESM.doc]

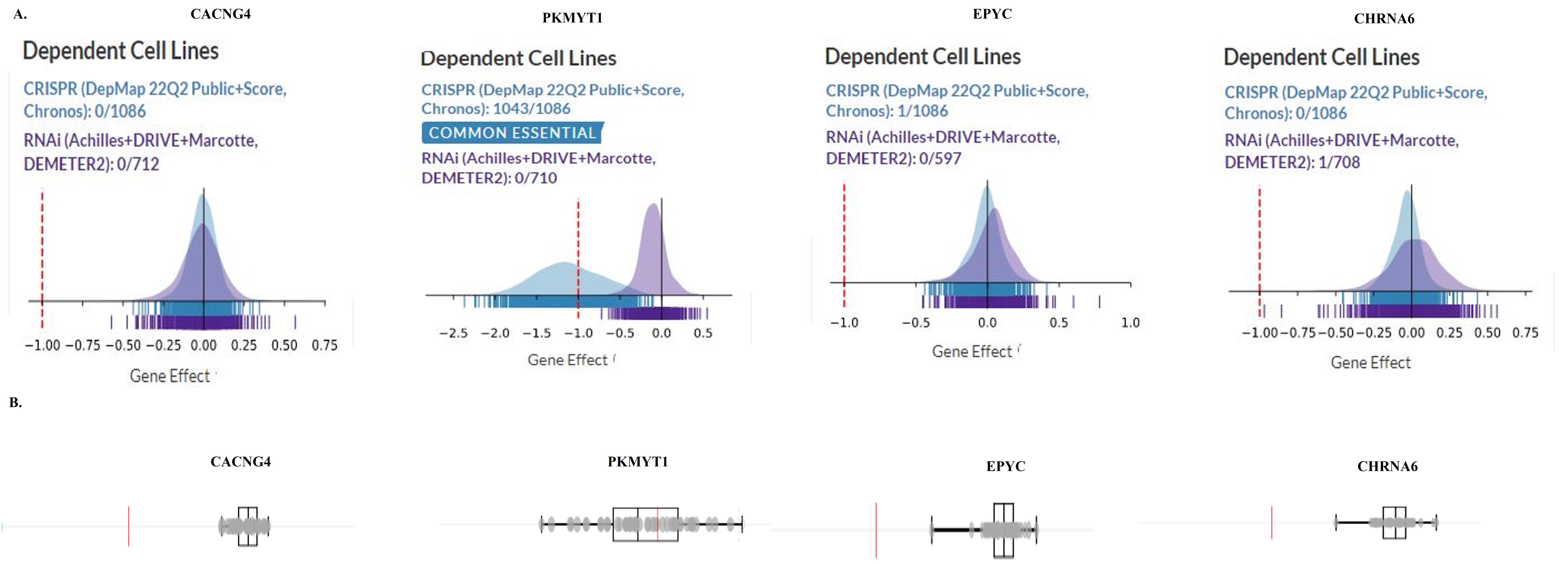


**Supplementary Fig.7**: **Tumor cell line dependency based on the DepMap tool**. A: The essential role of indicated genes (*CACNG4*, *PKMYT1*, *EPYC* and *CHRNA6*) in tumor cell line panels via DepMap, was established from CRISPR (blue) and RNAi (violet) databases. B: The Chronos dependence scores in breast cancer cells. A higher likelihood that the gene of interest is crucial in a particular cell line is indicated by a lower Chronos score. A gene is not essential if it has a score of 0 (dotted line); a value of 1 is like the average of all pan-essential genes (red line).
