# Supplementary material for: Unveiling promising breast cancer biomarkers: an integrative approach combining bioinformatics analysis and experimental verification
Source: BMC Cancer. 2024 Jan 31;24:155. doi: 10.1186/s12885-024-11913-7 (PMC10829368; doi:10.1186/s12885-024-11913-7)
Supplement: Supplementary file 16 — Additional file 16: Supplementary Table 4. Association of gene expression pattern of CACNG4,PKMYT1, EPYC, and CHRNA6 with clinico-pathological features in breast cancer patients. This file shows the expression patterns of these genes with age, ER status, PR status, HER2 status, TNM stages, histological grades and molecular subtypes. P value less than 0.05 considered to be statistically significant. SD: Significantly difference; NS: Non Significant; ER: Estrogen Receptor; PR: Progesterone Receptor; HER2: Human Epidermal Growth Factor Receptor 2; and TNM: Tumor, Node, Metastasis. [file 12885_2024_11913_MOESM16_ESM.doc]

| **Features** | | **Number (%)** | **Median**  **(PKMYT1)** | **P value** | **Median**  **(EPYC)** | **P value** | **Median**  **(CACNG4)** | **P value** | **Median**  **(CHRNA6)** | **P value** |
| --- | --- | --- | --- | --- | --- | --- | --- | --- | --- | --- |
| **Age (Years)** | ≥50 | 27 (67.5) | 1.565 | 0.0036  SD: Yes | 1.115 | 0.49  NS | 2.322 | 0.19  NS | 1.76 | 0.012  SD: Yes |
|  | <50 | 13 (32.5) | 0.830 |  | 1.321 |  | 2.785 |  | 0.91 |  |
| **LN Status** | Positive | 18 (45) | 1.033 | 0.54  NS | 1.543 | 0.6  NS | 2.319 | 0.7  NS | 1.18 | 0.58  NS |
|  | Negative | 22 (55) | 1.345 |  | 1.065 |  | 2.369 |  | 1.35 |  |
| **ER Status** | Positive | 18 (45) | 1.253 | 0.38  NS | 1.85 | 0.0001  SD:  Yes | 2.672 | 0.027  SD: Yes | 1.054 | 0.66  NS |
|  | Negative | 22 (55) | 1.234 |  | 0.96 |  | 2.319 |  | 1.25 |  |
| **PR Status** | Positive | 24 (60) | 1.253 | 0.83  NS | 1.376 | 0.040  SD: Yes | 2.369 | 0.27  NS | 1.155 | 0.18  SD: Yes |
|  | Negative | 16 (40) | 1.155 |  | 0.80 |  | 2.319 |  | 2.08 |  |
| **HER-2 Status** | Positive | 19 (47.5) | 1.548 | 0.023  SD: Yes | 1.265 | 0.27  NS | 2.352 | 0.6  NS | 1.6 | 0.0022  SD: Yes |
|  | Negative | 21 (52.5) | 0.828 |  | 1.115 |  | 2.324 |  | 0.9 |  |
| **TNM Stage** | Early Stages (І+ІІ) | 31 (77.5) | 1.259 | 0.94  NS | 1.016 | 0.031  SD: Yes | 2.348 | 0.4  NS | 2.38 | 0.51  NS |
|  | Late Stages (ІІІ+Іν) | 9 (22.5) | 1.208 |  | 1.892 |  | 2.464 |  | 2.6 |  |
| **Histological Differentiation** | Well/Moderate | 28 (70) | 1.231 | 0.24  NS | 1.293 | 0.89  NS | 0.41 | <0.0001  SD: Yes | 1.395 | 0.95  NS |
|  | Poor | 12 (30) | 1.276 |  | 0.999 |  | 2.255 |  | 1.276 |  |
| **Molecular Subtype** | Luminal | 28 (70) | 1.257 | NS | 1.376 | NS | 2.45 | NS |  | NS |
|  |  |  |  |  |  |  |  |  | 2.458  1.815 |  |
|  | HER-2 Overexpressed | 2 (5) | 1.364 |  | 0.64 |  | 1.81 |  |  |  |
|  | TNBC | 10 (25) | 0.80 |  | 0.87 |  | 2.259 |  | 2.259 |  |

**Supplementary Table 4: Association of gene expression pattern of *CACNG4*, *PKMYT1*, *EPYC*, and *CHRNA6* with clinico-pathological features in breast cancer patients.** Table 2 shows the expression patterns of these genes with age, ER status, PR status, HER2 status, TNM stages, histological grades and molecular subtypes. P value less than 0.05 considered to be statistically significant. SD: Significantly difference; NS: Non Significant; ER: Estrogen Receptor; PR: Progesterone Receptor; HER2: Human Epidermal Growth Factor Receptor 2; and TNM: Tumor, Node, Metastasis.
